# Knockdown of *NAT12/NAA30* reduces tumorigenic features of glioblastoma-initiating cells

**DOI:** 10.1186/s12943-015-0432-z

**Published:** 2015-08-21

**Authors:** Awais A. Mughal, Zanina Grieg, Håvard Skjellegrind, Artem Fayzullin, Mustapha Lamkhannat, Mrinal Joel, M. Shakil Ahmed, Wayne Murrell, Einar O. Vik-Mo, Iver A. Langmoen, Biljana Stangeland

**Affiliations:** Vilhelm Magnus Laboratory for Neurosurgical Research, Institute for Surgical Research and Department of Neurosurgery, Oslo University Hospital, Oslo, Norway; SFI-CAST-Cancer Stem Cell Innovation Center, Oslo University Hospital, Oslo, Norway; Norwegian Center for Stem Cell Research, Department of Immunology and Transfusion Medicine, Oslo University Hospital, Oslo, Norway; Laboratory of Neural Development and Optical Recording (NDEVOR), Department of Physiology, Institute of Basic Medical Sciences University of Oslo, Oslo, Norway; Institute for Surgical Research, Oslo University Hospital and Center for Heart Failure Research, University of Oslo, Oslo, Norway

**Keywords:** N-terminal acetyltransferases, NAA30, NAT12, hMAK3, Glioblastoma, Glioma, GICs, GSCs, NSCs and HIF1α

## Abstract

**Background:**

Glioblastoma (GBM) is the most common primary brain malignancy and confers a dismal prognosis. GBMs harbor glioblastoma-initiating cells (GICs) that drive tumorigenesis and contribute to therapeutic resistance and tumor recurrence. Consequently, there is a strong rationale to target this cell population in order to develop new molecular therapies against GBM. Accumulating evidence indicates that Nα-terminal acetyltransferases (NATs), that are dysregulated in numerous human cancers, can serve as therapeutic targets.

**Methods:**

Microarrays were used to study the expression of several NATs including *NAT12/NAA30* in clinical samples and stem cell cultures. The expression of *NAT12/NAA30* was analyzed using qPCR, immunolabeling and western blot. We conducted shRNA-mediated knockdown of *NAT12/NAA30* gene in GICs and studied the effects on cell viability, sphere-formation and hypoxia sensitivity. Intracranial transplantation to SCID mice enabled us to investigate the effects of *NAT12/NAA30* depletion in vivo*.* Using microarrays we identified genes and biochemical pathways whose expression was altered upon *NAT12/NAA30* down-regulation.

**Results:**

While decreased expression of the distal 3’UTR of *NAT12/NAA30* was generally observed in GICs and GBMs, this gene was strongly up-regulated at the protein level in GBM and GICs. The increased protein levels were not caused by increased levels of the steady state mRNA but rather by other mechanisms. Also, shorter 3’UTR of *NAT12/NAA30* correlated with poor survival in glioma patients.

As well, we observed previously not described nuclear localization of this typically cytoplasmic protein. When compared to non-silencing controls, cells featuring *NAT12/NAA30* knockdown exhibited reduced cell viability, sphere-forming ability, and mitochondrial hypoxia tolerance. Intracranial transplantation showed that knockdown of *NAT12/NAA30* resulted in prolonged animal survival.

Microarray analysis of the knockdown cultures showed reduced levels of *HIF1*α and altered expression of several other genes involved in the hypoxia response. Furthermore, *NAT12/NAA30* knockdown correlated with expressional dysregulation of genes involved in the p53 pathway, ribosomal assembly and cell proliferation. Western blot analysis revealed reduction of *HIF1*α, phospho-MTOR(Ser2448) and higher levels of p53 and GFAP in these cultures.

**Conclusion:**

*NAT12/NAA30* plays an important role in growth and survival of GICs possibly by regulating hypoxia response (HIF1α), levels of p-MTOR (Ser2448) and the p53 pathway.

**Electronic supplementary material:**

The online version of this article (doi:10.1186/s12943-015-0432-z) contains supplementary material, which is available to authorized users.

## Background

Glioblastoma multiforme (GBM) is the most common and the most aggressive primary brain cancer. The average annual age-adjusted incidence rate is ~3 per 100.000 person-years [1]. Despite multimodal therapy and inclusion of temozolomide, the overall survival remains dismal [2, 3]. This grim scenario mandates a pivotal shift in our approaches to develop new therapy as well as understanding the underlying disease mechanisms. During the last decade the GBM field has shown interest in a subgroup of cells harbored within these tumors with stem cell characteristics [4–6] that forms invasive tumors, similar to the tumor of origin, upon orthotopic xenotransplantation [7]. These cells are commonly referred to as glioblastoma-initiating cells (GICs) and deemed important for the characteristics of GBM. They possess enhanced invasive properties [8, 9], promote tumor angiogenesis [10–12] and are resistant to irradiation [13] and chemotherapy [14, 15]. Thus, there is a strong rationale for validating potential molecular targets in GICs.

Dysregulation of co- or post-translational protein modifications is a trait of many human cancers. Nα- or N-terminal (Nt-) acetylation is one of the most common covalent modifications of all soluble human proteins [16] and occurs predominantly co-translationally [17]. N-terminal acetyltransferases (NATs) or Nα-acetyltransferases (NAAs) are arranged in complexes and believed to target ~80-90 % of all soluble human proteins [17, 18]. Six NAT complexes have been identified in humans, NAT-A – NAT-F and each complex consists of one catalytic subunit and auxiliary subunit(s) [16]. With an increasing body of knowledge about their diverse functions, substrates and downstream targets, NATs are emerging as potential targets in several cancers [16]. Knockdown studies targeting NATs in various malignancies including colon [19], thyroid [20] and hepatocellular cancers [21] have shown that depletion of the catalytic subunit causes a less aggressive phenotype with reduced cell proliferation and/or increased apoptosis.

NAT12/NAA30 is the catalytic subunit of the NAT-C complex [22]. Knockdown of each of the NAT-C subunits, led to p53-dependent apoptosis in HeLa and colon carcinoma cell lines with the strongest phenotype observed with depletion of *NAT12/NAA30* [23]. The latter study also pointed out mammalian target of rapamycin (MTOR) as a substrate for NAT-C*.* Another report also suggested TOR as a target of NAT-C activity [24].

In the present study we investigated the expression of *NAT12/NAA30* in GBM tissue samples, GICs, normal brain tissue, and neural stem cells (NSCs) from the adult human brain as well as in a neural fetal cell line (NFCs). Using immunolabeling, we revealed a hitherto undescribed nuclear localization of NAT12/NAA30 protein. To study the function of *NAT12/NAA30,* we performed gene knockdown using RNA interference (RNAi) technology. Knockdown of *NAT12/NAA30* resulted in markedly reduced cell viability and sphere-forming ability of GICs. To study genes and pathways downstream of *NAT12/NAA30*, we used microarray analysis and western blot. This enabled us to identify several pathways such as p53, ribosomal assembly, hypoxia response and cell proliferation regulated by *NAT12/NAA30.* Furthermore, we documented a reduction of phospho-MTOR (Ser2448) and increased levels of p53 and glial fibrillary acidic protein (GFAP) in the knockdown cultures. We show that intracranial transplantations into severe combined immunodeficient (SCID) mice of GICs featuring *NAT12/NAA30* knockdown, resulted in a significant prolongation of animal survival compared to controls.

## Results

### Expression of *NAT12/NAA30* in brain tissues, GBM, NSCs, GICs and NFCs

To investigate the expression of *NAT12/NAA30* in GBM tumor biopsies, normal human brain, NSCs, GICs and NFCs we used microarrays, real-time quantitative reverse-transcription PCR (qPCR), western blots, immunolabeling and public data mining.

*Microarray analysis* was performed on GIC cultures from seven patients, ten NSC cultures from five patients, two GBM biopsies, two normal brain tissue samples and one NFC culture. NSC cultures were isolated from four brain regions: the subventricular zone (SVZ), hippocampus (HPC), white- and grey matter (WM and GM respectively). Normal tissues were from WM and GM.

**Fig. 1:**
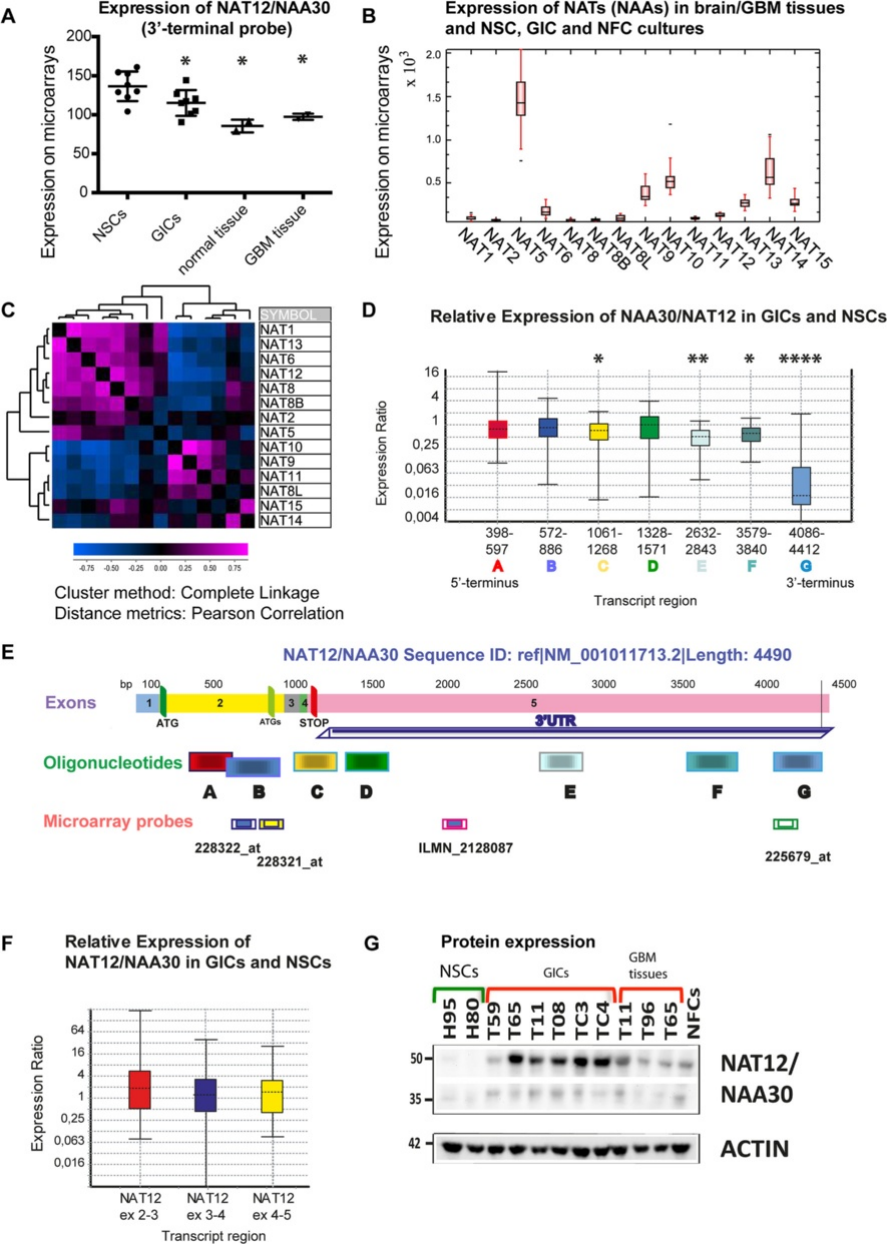
Expression of NAT12/NAA30 in GIC and NSC cultures, NFCs, and in GBM and normal brain tissues. **a**, Expression of *NAT12/NAA30* is analysed by microarray (3’UTR Reporter = ILMN_2128087). Expression is 1.2 fold higher in NSCs than in GICs (*p* = 0.0281). P values were calculated using the Mann Whitney test. Due to the small sample size the values were not calculated for tissues and NFCs. **b**, Box and whiskers plot showing the expression of the selected NATs in all samples as measured by microarrays. **c**, Hierarchical clustering (with distance matrix) of the expression values for selected NAT genes in all samples. **d**, Expression of *NAT12/NAA30* in GIC cultures was calculated using qPCR and the seven sets of oligonucleotides whose positions are delineated in (**e**). NSCs were used as a control. P values in this experiment were as following p(A) = 0.641, p(B) = 0.532, p(C) = 0.033, p(D) = 0.268, p(E) = 0.007, p(F) = 0.012, and p(G) = 0. The bottom and top of each box indicate the 25th and 75th percentile (the lower and upper quartiles, respectively), and the band near the middle of the box is the 50th percentile (the median). The ends of the whiskers represent the minimum and maximum of all the data. Additional details of the statistical analysis can be found in Additional file 4. **e**. Detailed architecture of the reference sequence of the *NAT12/NAA30* transcript (NM_001011713.2) and details on probes and oligonucleotide positions. **f**, Relative expression of the coding regions of *NAT12/NAA30* in GICs compared to NSCs as measured with Taqman probes. P values for this analysis were (0.266, 0.970 and 0.672). Additional details of the statistical analysis can be found in Additional file 4. **g**. Expression of the NAT12/NAA30 protein in NSC and GIC cultures, GBM and NFCs

*NAT12/NAA30* expression (measured with the 3’ terminal ILMN_2128087 reporter) was moderately but significantly higher in NSC compared to GIC cultures (Fig. 1a). It was also higher in the cell cultures compared to tissues (Additional file 1: Figure S1). We also analyzed the expression of several other NATs in the same set of microarrays and found that *NAT5, NAT14, NAT10* and *NAT9* were among the most abundant NATs in the analyzed cell cultures and tissues (Fig. 1b). *NAT12/NAA30* was among the relatively lowly expressed NATs (Fig. 1b). K-means clustering (Additional file 2: Figure S2) showed that *NAT12/NAA30* was co-expressed with *NAT1, NAT13* and *NAT5*. Hierarchical clustering with distance matrix using Pearson correlation as a distance measure (Fig. 1c) revealed that *NAT12/NAA30* was highly co-expressed with *NAT8, NAT8B, NAT1, NAT6, NAT13,* and co-expressed with *NAT2* and *NAT5* (Fig. 1c). Several NATs from this group had significantly lower expression in GIC than in NSC cultures (*NAT12, NAT6, NAT8* and *NAT8B*). Several other NATs such as *NAT9, NAT10, NAT14* and *NAT15* were expressed to a similar degree in GIC and NSC cultures. *NAT8L* was the only NAT that was significantly up-regulated in GIC cultures and GBM (Additional file 1: Figure S1).

*qPCR analysis* was used to validate the microarray results. We used seven sets of oligonucleotides that covered both coding (A-C) and non-coding (D-G) regions of the gene (Fig. 1d-e, Additional file 3) to investigate *NAT12/NAA30* expression in seven GIC cultures, two NSC cultures and one NFC culture.

qPCR revealed that the relative expression of the coding regions of *NAT12/NAA30* was very variable between different GIC cultures. However, the average expression values were similar in GIC and NSC cultures as shown by qPCR with oligonucleotides corresponding to the coding regions of the gene (Fig. 1d; Oligonucleotide sets A and B, Additional file 4) and Taqman probes corresponding to the exons (Fig. 1f).

Interestingly, when oligonucleotides corresponding to the 3’UTR (C, E, F and G) were used (Fig. 1e), qPCR analysis showed that *NAT12/NAA30* expression was reduced in GIC cultures (Fig. 1d). We observed almost complete absence of expression (97 % reduction) in GIC cultures when using oligonucleotide set G, which corresponds to the most distal region of the 3’UTR (Fig. 1d, Additional file 4).

*Western blot.* NAT12/NAA30 protein expression in NSC and GIC cultures, GBM tissues and NFCs was investigated using western blots (Fig. 1g). We detected a stronger band at around 50 kDa in the GICs although the expected sizes of the two isoforms of the protein were 35 and 39 kDa (Fig. 1g). This might indicate either protein modification or the presence of a different isoform in the GICs. Bands of comparable sizes were detected using another antibody and recombinant protein confirming that the correct bands were detected (results not shown). Western blot analysis revealed that NAT12/NAA30 expression in GIC cultures was significantly higher than in NSC cultures (Fig. 1g) although this was not observed at RNA level (Fig. 1a, d and f).

*Survival analysis.* Public database mining was done using data from the REMBRANDT database (https://caintegrator.nci.nih.gov/rembrandt/) where 3 probes were used to measure the expression of *NAT12/NAA30* covering different parts of the transcript (Fig. 1e; microarray probes ending with “_at”).

Interestingly, the 3’UTR of *NAT12/NAA30* seemed to be universally down-regulated in glioma tissue samples. This was in accordance with our data obtained by both microarray analysis and qPCR (Fig. 1a and d). Glioma patients with decreased expression of the distal 3’UTR end of the *NAT12/NAA30* transcript (Probe: 225679_at, Fig. 1e) had significantly shorter survival (Additional file 5: Figure S3A), while the increased expression of the coding regions of this gene (Probes: 228322_at, 228321_at, Fig. 1e) did not seem to correlate with patient survival (Additional file 5: Figure S3B-C). For comparison, we also checked the association between the expression levels of other NATs in glioma tissues and patient survival. Increased expression of each of *NAT1, NAT2* and *NAT10* correlated negatively with patient survival in the group of “all gliomas” patients (Additional file 5: Figure S3D-F).

**Fig. 2:**
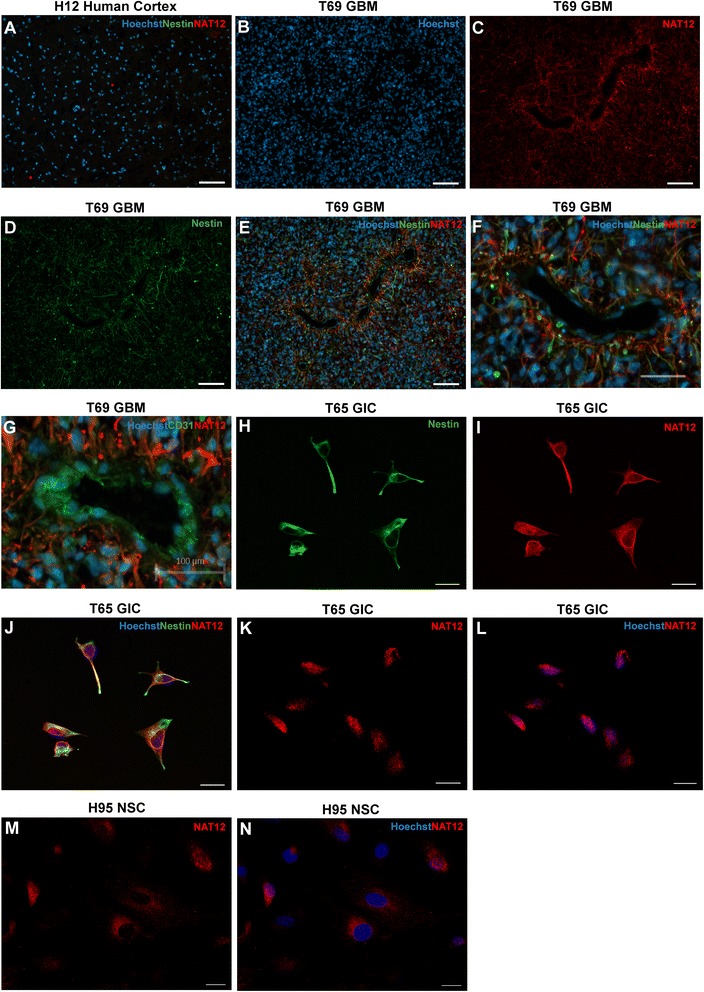
Immunolabeling with anti-NAT12/NAA30 antibody performed on GIC and NSC cultures, GBM tissues and brain tissue. **a**, Immunolabeling of a biopsy from normal human cortex shows no expression of NAT12/NAA30 (red) and nestin (NES, green). **b**, Hoechst staining showing high nuclear density in a GBM biopsy. **c**, Immunolabeling of NAT12/NAA30 (red) in a GBM biopsy. **d**, Immunolabeling of NES (green) in a GBM biopsy. **e**, Extensive staining of both NAT12/NAA30 (red) and NES (green) in a GBM tissue specimen. **f**, NAT12/NAA30 positive cells are abundant around vessels. **g**, Co-staining of NAT12/NAA30 (red) and CD31 (green) confirms that NAT12/NAA30 cells are located in the perivascular niche of the tumor. Scale bar is 100 μm. **h**-**j**, Confocal images of NAT12/NAA30 immunolabeling show that NES (green) and NAT12/NAA30 (red) is co-expressed in GICs. NES is present in the cytoplasm and NAT12/NAA30 is also found in the nuclei. Scale bar is 20 μm. **k**-**n**, Confocal images showing cellular expression pattern of NAT12/NAA30 (red) in GIC– and NSC cultures. NAT12/NAA30 (red) is predominantly located in the cytoplasm of NSCs but is consistently present in the nuclei in GIC cultures depicted by the overlap with Hoechst (blue). Scale bar is 20 μm

*Immunolabeling.* We tested the expression of the NAT12/NAA30 protein in three GIC and three NSC cultures, and in frozen sections from two GBMs and two normal brain tissues (Fig. 2 and Additional file 6: FigureS4). NAT12/NAA30 protein was barely detectable in samples from normal brain (Fig. 2a, Additional file 6: Figure S4A) but it was highly up-regulated in the GBM biopsies (Fig. 2c, Additional file 6: Figure S4B). GBM tissue sections were co-stained with anti-NAT12/NAA30 and anti-nestin antibodies (Fig. 2d). This revealed a cell population expressing both proteins (Fig. 2e). The cells expressing both nestin and NAT12/NAA30 were detected frequently in GICs (Fig. 2h-j). Interestingly, in the GBM sections, NAT12/NAA30 expressing cells were located in the perivascular spaces (Fig. 2f). The same staining pattern was confirmed with another antibody against NAT12/NAA30 (results not shown). The observation that NAT12/NAA30 was highly expressed in cells surrounding blood vessels, was verified by co-staining with anti-CD31 antibody (Fig. 2g).

NAT12/NAA30 was previously detected in the cytoplasm of various eukaryotic cells [23–26]. However, our immunolabeling revealed an hitherto unreported nuclear localization of NAT12/NAA30. The nuclear localization was consistently detected in all tested GIC cultures but was more sporadic in the NSC cultures (Fig. 2k-n, Additional file 6: Figure S4C-F).

### *NAT12/NAA30* knockdown

**Fig. 3:**
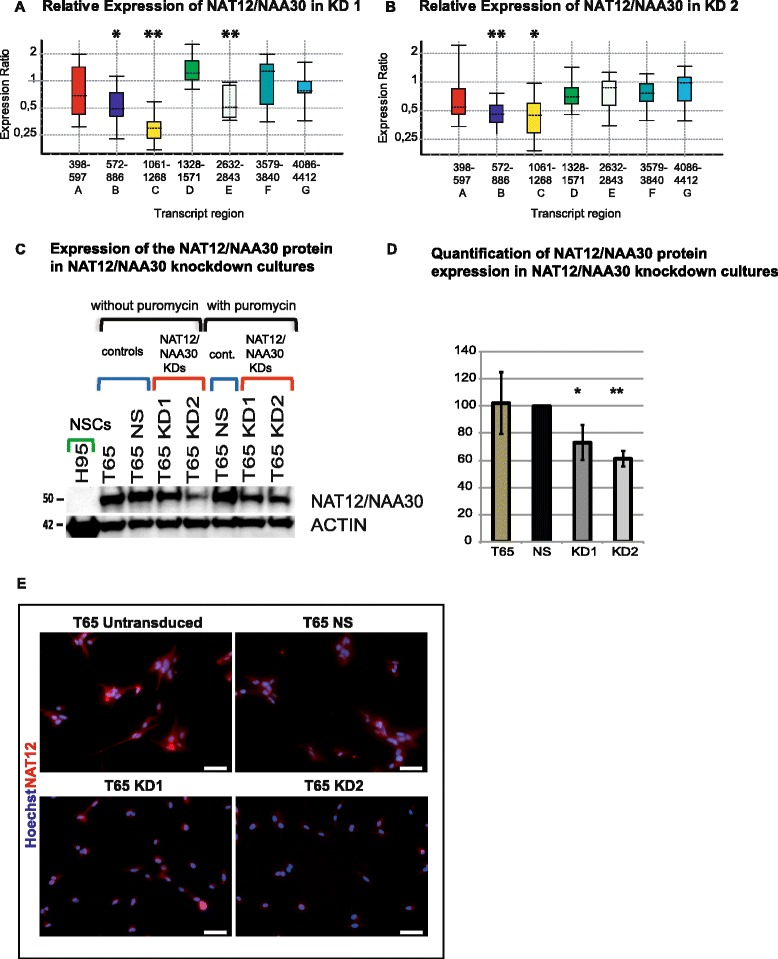
Confirmation of *NAT12/NAA30* gene knockdown at RNA and protein level. **a**-**b**, Box and whiskers plot of gene expression as measured by qPCR. The bottom and top of each box indicate the 25th and 75th percentile (the lower and upper quartiles, respectively), and the band near the middle of the box is the 50th percentile (the median). The ends of the whiskers represent the minimum and maximum of all the data. **a**, Relative expression of *NAT12/NAA30* in KD1 culture was analysed using seven sets of oligonucleotides designed to cover the whole transcript (for oligonucleotide position see Fig. 1e). Relative expression (RE) calculated by normalization to the NS culture and the statistical parameters. P values in this experiment were as follows p(A) = 0.35, p(B) = 0.011, p(C) = 0.001, p(D) = 0.146, p(E) = 0.001, p(F) = 0.915, and p(G) = 0.367. Asterisks correspond to p values and indicate level of significance. * = (p ≈ 0.01-0.05), ** = (p ≈ 0.001-0.01), *** = (p ≈ 0.0001-0.001), **** = (*p* < 0.0001). Additional details of the statistical analysis can be found in Additional file 4. **b**, The same for KD2 culture. P values in this experiment were as follows: p(A) = 0.368, p(B) = 0.002, p(C) = 0.019, p(D) = 0.099, p(E) = 0.256, p(F) = 0.167, and p(G) = 0.61. Asterisks correspond to p values and indicate level of significance. Additional details of the statistical analysis can be found in Additional file 4. **c**, Western blot showing knockdown of NAT12/NAA30 protein in KD1 and KD2. **d**, Quantification of the protein expression from western blots (average of three blots with two different antibodies) showing relative expression of NAT12/NAA30 protein in knockdown cultures KD1 and KD2 compared to the controls, NS and T65 untransduced. **e**, Immunolabeling showing expression of NAT12/NAA30 (red) in the two controls, T65 untransduced and T65 NS and reduced protein levels in KD1 and KD2. Scale bar is 50 μM

In order to study the functional relevance of *NAT12/NAA30* in GICs, we established short hairpin RNA (shRNA)-mediated knockdown of *NAT12/NAA30*. We transduced the GIC culture T65 with lentiviruses harboring two different shRNA sequences and obtained transgenic cell cultures (KD1 and KD2). Non-silencing (NS) shRNA was used as a control. Knockdown efficiency was assessed with qPCR, western blot and immunolabeling to confirm depletion of *NAT12/NAA30* transcript and -protein. The gene knockdown efficiency was measured with oligonucleotides corresponding to the coding (A-C) and non-coding (D-G) regions of the gene (Fig. 1e, Fig. 3a-b). Relative expression of *NAT12/NAA30* was reduced with 60 ± 14 % in KD1 (Fig. 3a) and 56 ± 3 % in KD2 (Fig. 3b). For this calculation we used average values for two sets of oligonucleotides (B and C) corresponding to the coding regions of the gene (Fig. 1e, Additional file 4). Transcripts corresponding to the 3’UTR were predominantly not down-regulated in knockdown cultures KD1 and KD2 as shown by qPCR using oligonucleotides D-G (Fig. 3a-b).

Western blot showed reduction of NAT12/NAA30 protein levels in both KD1 and KD2 cultures (Fig. 3c). To quantify the efficiency of knockdown at the protein level, we used three western blots and two antibodies. We measured an average reduction of 27 ± 13 % in KD1 and 40 ± 6 % in KD2 (Fig. 3d).

Immunolabeling confirmed reduced protein expression in GICs featuring *NAT12/NAA30* gene knockdown compared to both untreated control and NS (Fig. 3e). This was also confirmed with another antibody against NAT12/NAA30 (results not shown).

### Knockdown of *NAT12/NAA30* in GICs reduces cell viability and sphere-forming ability

We investigated the functional effects of the *NAT12/NAA30* knockdown in GICs by measuring cell viability and sphere-forming ability in NS, KD1 and KD2 cultures.

**Fig. 4:**
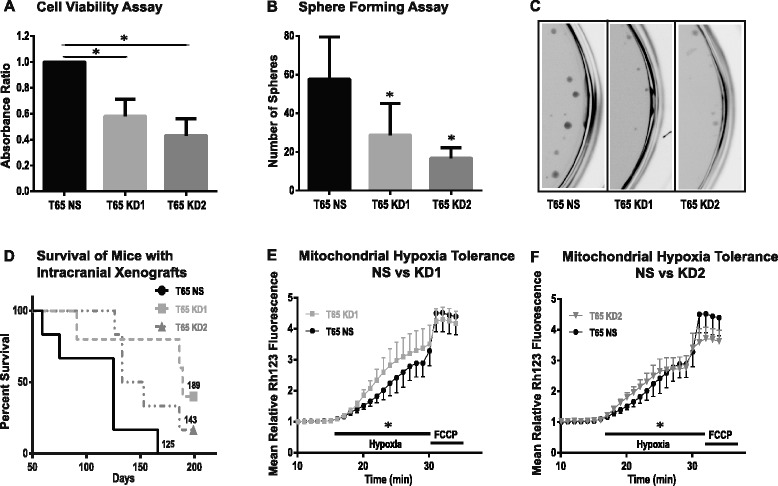
Functional effects of knockdown of *NAT12/NAA30* in GIC cultures. **a**, Calorimetric XTT assay was used for the quantitative assessment of cell viability. Analysis demonstrated a significantly lower percentage of viable cells in the knockdown cultures KD1 and KD2 compared to the NS control. Error bars indicate standard deviation (n = 3). Asterisks signify p-values < 0.05. **b**, A sphere-forming assay was used to assess the ability of the GICs to form colonies. Sphere number was decreased by 50-71 % in the knockdown cultures compared to the NS control. Error bars indicate standard deviation of three independent experiments. Asterisks signify p-values < 0.05. **c**, Images of wells obtained from a sphere-forming experiment showing far less spheres in KD1 and KD2 than NS control. **d**, Kaplan-Meier survival plot demonstrates that control animals (NS) died much earlier than GIC cultures (KD1 and KD2) featuring *NAT12/NAA30* knockdown. Median survival in days for each group is shown in the plot. **e**-**f**, GIC cultures featuring *NAT12/NAA30* knockdown (KD1 and KD2) had decreased mitochondrial hypoxia tolerance. Error bars indicate standard deviation of more than 20 cells from at least four independent experiments (p-value < 0.01)

Values obtained by the XTT assay for the knockdown cultures were normalized to the NS control. The number of viable cells was reduced to 58 ± 8 % for KD1 (*p* = 0.0314; unpaired *t*-test with Welch’s correction) and 43 ± 8 % (*p* = 0.0172) for KD2 compared to the NS control. This demonstrated a significant reduction in cell viability of GICs featuring *NAT12/NAA30* knockdown (Fig. 4a).

The number of spheres formed from cells seeded at a low density (500 cells per well) was 29 ± 7 (*p* = 0.0472) in KD1 and 17 ± 2 (*p* = 0.121) in KD2 compared to 58 ± 10 in the NS control (Fig. 4b-c). Our results thus indicate that the *NAT12/NAA30* knockdown reduced sphere-forming ability by 50–71 %. The sphere-size was, however, not affected (results not shown).

### Knockdown of *NAT12/NAA30* in GICs prolonged survival of intracranially xenografted mice

To investigate the effect of *NAT12/NAA30* knockdown on GIC growth and tumor formation in vivo we used a robust orthotopic xenograft model [27, 28]. Cells from the knockdown cultures KD1 and KD2, and the NS control culture were transplanted into the right hemisphere of SCID mice (*n* = 6 for each group).

Median survival for mice transplanted with NS cells was 125 days versus 189 for KD1 (*p* = 0.0192; Log-rank test) and 143 for KD2 (*p* = 0.0385) (Fig. 4d). All 6/6 animals in the NS control group died whereas 2 animals in KD1 group and 1 in KD2 group did not show any sign of distress at the end of the observation period of 200 days. Representative cryosections stained with Hematoxylin and Eosin (HE) from the xenograft brains are shown in Additional file 6: Figure S4G.

### Knockdown of *NAT12/NAA30* in GICs decreased mitochondrial hypoxia tolerance

The ability to tolerate hypoxic conditions is a well known trait of GICs and it contributes to the tumor cells’ evasion of apoptosis [29]. It has previously been shown that *NAT12/NAA30* is involved in regulation of apoptosis [23]. Mitochondrial depolarization is an early step in programmed cell death. To test if down-regulation of *NAT12/NAA30* influences mitochondrial properties, such as mitochondrial hypoxia tolerance, we compared the mitochondrial response to acute severe hypoxia in NS and knockdown cultures, KD1 and KD2.

Baseline recordings of mitochondrial membrane potential (ΔΨ_m_) were stable in all cells. Increase in Rh123 fluorescence, indicating mitochondrial membrane depolarization, was seen in all three groups within 3–5 min of hypoxia. Compared to the NS control, there was a steeper increment of the fluorescent signal in KD1 and KD2 (Fig. 4e-f; KD1 and KD2 vs. NS, both *p* < 0.01, one-way ANOVA). The remaining ΔΨ_m_ was released upon addition of the protonophore FCCP. The FCCP response of the control cells was almost twice that of the *NAT12/NAA30* knockdown cells (relative fluorescence increase: NS 1.63; KD1 0.92; KD2 0.95) indicating that the remaining mitochondrial membrane potential after hypoxia was higher in the NS control cells than in KD1 and KD2. The difference was, however, not statistically significant (Fig. 4e-f; *p* = 0.07, two-way ANOVA).

### Identification of genes and biological pathways downstream from *NAT12/NAA30*

To identify genes and biological pathways downstream from *NAT12/NAA30,* we used microarrays, qPCR and western blot.

Microarray analysis showed that 661 Illumina probes identified transcripts that were differentially regulated (1.5-fold change) in GICs featuring *NAT12/NAA30* knockdown (Additional file 7). Of these 464 were recognized and analyzed by the DAVID functional annotation tool [30, 31]. This analysis revealed that the *NAT12/NAA30* knockdown had a significant effect (*p* = 3.00E-05) on the expression of genes coding for ribosomal proteins (Additional file 7). Many of these were directly involved in protein translation (Additional file 7). KEGG-pathway analysis identified three significantly dysregulated pathways: 1) Ribosome (13 genes, *p* = 5.6E-06), 2) p53 pathway (8 genes, *p* = 3.1E-03) and 3) Sphingolipid metabolism (6 genes, *p* = 4.8E-03) (Additional file 8: Figure S5–S7 and Additional file 7).

**Fig. 5:**
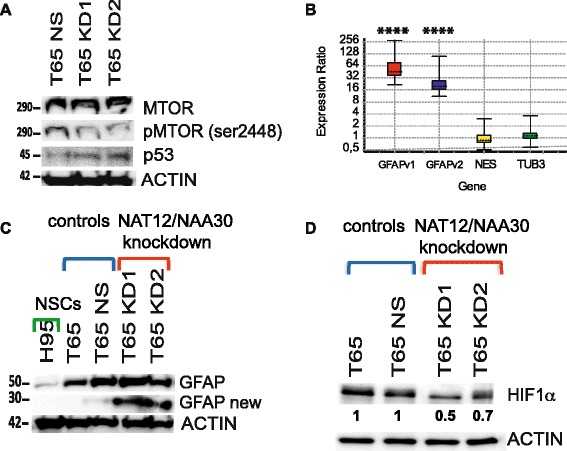
Analysis of genes and pathways downstream from *NAT12/NAA30*. **a**, Western blot showing the expression of MTOR, pMTOR-Ser2448, p53 and ACTB (ACTIN) proteins. **b**, Relative expression of *GFAP*, *NES* and *TUBB3* were measured by qPCR (normalized to NS). P values in this experiment were as follows: p(GFAPv1) = 0, p(GFAPv2) = 0, p(NES) = 0.926, p(TUB3) = 0.348. Asterisks correspond to p values and indicate level of significance. **** = (*p* < 0.0001). Additional details of the statistical analysis can be found in Additional file 4. **c**, Western blot showing the protein expression of NAT12/NAA30, GFAP and ACTB in the knockdown cultures KD1 and KD2, NS culture, GIC culture T65 and NSCs. **d**, Western blot showing the expression of HIF1a and ACTB in both knockdown cultures and controls

A more detailed analysis of the p53 pathway revealed that the expression of eight genes in this pathway was differentially regulated in the *NAT12/NAA30* knockdown cultures. These genes were involved in apoptosis, cell cycle arrest and DNA repair (Additional file 8: Figure S6 and Additional file 7). Former studies have shown that knockdown of the NAT-C complex leads to reduced proliferation and p53-dependent cell death in human cancer cell lines [23]. Starheim et al. reported increased levels of total p53, phospho-p53 (Ser37) and apoptosis in HeLA cells featuring *NAT12/NAA30* knockdown [23]. In our knockdown cultures, western blot showed a slight increment in the total p53 (Fig. 5a), while the levels of the phospho-p53 (Ser37) were not altered (Additional Additional file 9: Figure S8A). We could not detect any significant changes in apoptosis in the knockdown cultures (results not shown).

Ten genes involved in the response to hypoxia (*p* = 6.80E-03; GOTERM_BP_FAT: response to hypoxia) were differentially regulated in the knockdown cultures (Additional file 10: Figure S9 and Additional file 7). Further analysis of the hypoxia response pathway showed that the hypoxia induced factor 1α (*HIF1*α), potassium large conductance calcium-activated channel *(KCNMA1)* and the integrin receptor (*ITGA2)* (involved in cell survival and growth via the PI3K pathway) were down-regulated in the knockdown cultures. HIF1α is one of the central regulators of transcriptional control in tumor cells’ response to hypoxic conditions. In GBM, it is regarded as a key player in tumor progression and an important factor for the maintenance of GIC phenotype [32, 33]. Therefore, it was of great interest to analyze the expression of HIF1α protein in the *NAT12/NAA30* knockdown cultures. Notably, western blot showed that the levels of HIF1α were decreased in the knockdown cultures compared to the controls (Fig. 5d), thus confirming the microarray results. The levels of KCNMA1 protein were, however, not altered in the knockdown cultures (Additional file 9: Figure S8B). Among the up-regulated genes were the growth inhibiting factor metallothionein 3 *(MT3)*, *CD24*, chemokine receptor type 4 (*CXCR4*) and chemokine ligand 2 (*CCL2*) (Additional file 10: Figure S9 and Additional file 7).

Among other genes differentially expressed in the *NAT12/NAA30* knockdown cultures, were groups of genes coding for: a) proteins involved in organization of nucleosomes (11 genes, *p* = 1.1E-04), b) cell proliferation (22 genes, *p* = 3.50E-03) and c) protein kinase activity (22 genes, *p* = 1.8E-04) (Additional file 7).

Previous studies have identified MTOR as a potential substrate for the NAT-C complex [23] and a downstream target of NAT-C activity [24]. Microarray analysis of the GIC cultures featuring *NAT12/NAA30* knockdown, showed increased expression of insulin-like growth factor binding protein 3 *(IGFBP3)*, the inhibitor of the IGF1/MTOR pathway as compared to NS controls (Additional file 8: Figure S6). Western blot showed that total MTOR was not changed while the levels of phospho-MTOR (Ser2448) were reduced in the knockdown cultures (Fig. 5a). Western blot analysis of selected important factors in the mTOR/AKT pathway is provided in Additional file 9: Figure S8C.

One of the key features of GICs is the high expression levels of the pluripotency marker nestin (NES) and that they can be differentiated into a neuronal or glial lineage [5, 27]. Upon differentiation the proliferative capacity of these cells is lost while the levels of the neuronal marker β3-tubulin (TUBB3) and the astrocytic marker GFAP increase [27]. As we have shown *NAT12/NAA30* knockdown reduced GIC proliferation (Fig. 4a-b). Thus, we wanted to investigate whether knockdown of *NAT12/NAA30* altered the expression levels of the aforementioned markers towards a more mature cell type. Interestingly, in both knockdown cultures *GFAP* was highly up-regulated while the expression of *NES* and *TUBB3* remained unchanged as shown by qPCR analysis (Fig. 5b). However, the expression of nestin at the protein level was reduced in the knockdown cultures (Additional file 9: Figure S8B). Western blot showed a so far undescribed variant of GFAP (30 kDa) highly upregulated in the knockdown cultures (Fig. 5c).

We have tested the expression of stem cell related genes in the *NAT12/NAA30* knockdown cultures. Flow cytometric analysis showed that the CD133+ population was depleted in both knockdown cultures compared to the NS control (Additional file 11: Figure S10). We did not detect the expression of CD15 in T65 (results not shown). We also evaluated the expression of *NANOG*, *POU5F1/OCT4*, *SOX2* and *MYC* using microarrays. None of these genes were differentially regulated at the mRNA level in the knockdown cultures (Additional file 9: Figure S8C). However, the levels of SOX2 protein were slightly decreased in the two knockdown cultures as shown by western blot (Additional file 9: Figure S8B).

Western blot analysis further revealed that the levels of active β-catenin (active CTNNB1/ABC), phospho-p70 S6 kinase (Thr389), phospho-STAT3 (Ser727), GLI1 and CCND1 and several other proteins were only slightly affected or not altered in the knockdown cultures (Additional file 8: Figure S8A-B).

## Discussion

GBM remains one of the most aggressive human cancers. Accumulating evidence supports the role of GICs in disease progression, tumor cell invasion and the tumor’s notorious resistance to chemo- and radiotherapy. Consequently, new molecular therapies against GBM would also need to target this cell population.

Recently, protein Nt-acetylation has been implicated in cancer development [16]. In the present study we analyzed the expression of several NATs in GIC cultures and GBM tissues showing which of these are co-expressed and thus might be functionally redundant (Fig. 1b-c and Additional file 4: Figure S2). We also show that the increased expression of several NATs in GBM tissues is associated with poor patient survival and that these enzymes may be relevant as new potential therapeutic targets. To further reinforce the validity of our study we used NSC cultures isolated from several different regions of the brain as controls. We identified NATs whose expression was significantly down- or up-regulated in GBM and GICs (Additional file 3: Figure S1).

By detailed expression analysis of *NAT12/NAA30* in tissues and cell cultures (GIC and NSC cultures, and NFCs), we detected the presence of alternative transcript(s) that had 3’UTR regions of variable length (Fig. 1d). We have demonstrated that these were differentially regulated in GICs compared to NSCs. Especially the distal 3’UTR of *NAT12/NAA30* was strongly down-regulated in GBM and GIC cultures as shown by two independent methods (microarray and qPCR) (Fig. 1a and d). Moreover, public database mining revealed that the decreased expression of the distal 3’UTR region correlated with a shorter survival of glioma patients (Additional file 5: Figure S3A). Several studies have shown that mRNA isoforms with differences in their 3’UTRs have different stability and translational activity [34–36]. In yeast it was found that shorter mRNAs more frequently formed a closed-loop structure that enhanced protein translation [37]. Mayr et al. studied 27 cancer cell lines from different tissues and showed that shortening of the 3’UTR in the tested mRNAs seemed to be important for activation of oncogenesis [38]. Shortening of the 3’UTRs resulted in enhanced translation in some of these cancer cell lines. More importantly, shorter mRNAs had greater stability and produced more protein [38]. Another study reported that shortening of 3’UTRs correlated with poor patient survival in breast and lung cancer [39]. To our knowledge we are the first to report the existence of an analogous mechanism associated with GBM and GICs. In the present work we show that the up-regulation of NAT12/NAA30 at the protein level in GICs is not caused by the increased levels of the steady state mRNA but rather by other mechanisms such as shortening of the 3’UTR and possibly greater stability of mRNA. Other processes involved in the complex regulation of *NAT12/NAA30* expression might be alternative splicing, alternative poly-adenylation sites (Stangeland, unpublished), and the presence of regulatory and/or coding sequences within the 3’UTR.

Human NAT12/NAA30 is known to be present in the cytoplasm [23–26]. Our data show that it is also located in the nuclei of GICs and some NSCs (Fig. 2i and k). The transport of proteins from the cytosol to the nucleus is a tightly regulated process facilitated by importins [40]. NAT12/NAA30 contains a nuclear localization signal (RRGYIAMLAVDSKYRRN at 243 according to pSORT II, http://psort.hgc.jp/) that confers a high probability of being imported to the cell nuclei. It has also been reported that 3’UTRs of other genes contain regulatory regions that influence nuclear export and subcellular localization [41]. The observed differential subcellular localization of NAT12/NAA30 in the nucleus is novel and might imply an additional role for NAT12/NAA30. Another member of the NAT family, NAT-D, is known to acetylate histones H2A and H4 [42]. Whether NAT12/NAA30 protein can have a similar role remains to be shown.

Furthermore, evaluating the staining pattern of NAT12/NAA30 in GBM biopsies led to an interesting observation (Fig. 2b-f). NAT12/NAA30 was found to be expressed predominantly in cells surrounding blood vessels. In this perivascular region the GBM cells have a much stronger expression of nestin than in other parts of the tumor specimen. Co-staining with these two markers showed a large degree of overlap in the GBM cells around blood vessels. Recently, it was shown that nestin regulates stemness, cell growth and invasion in GBMs [43]. The authors reported that the overexpression of *NESTIN* increased cell growth, sphere formation and cell invasion while depletion of *NESTIN* resulted in decreased expression of stem cell markers. The expression of nestin has been found to be an independent prognostic factor in glioma patients [44]. Since GICs are believed to reside in the perivascular niche of the tumor [10–12], our immunolabeling might indicate that NAT12/NAA30 is predominantly expressed in an immature cell types in GBM.

RNAi-mediated gene silencing of *NAT12/NAA30* enabled us to obtain a stable knockdown at both transcript and protein level as determined by qPCR, western blot and immunolabeling (Fig. 3). NATs are evolutionarily highly conserved [17, 22]. The partial knockdown we obtained might be explained by selection mechanisms having enabled GICs to avoid the lethality that would result from total *NAT12/NAA30* knockdown. Nevertheless, functional analysis of the knockdown cultures revealed a clear phenotype with restricted cell growth in vitro and in vivo (Fig. 4a-d). Reduced sphere-forming ability in the knockdown cultures was stable across passages. This finding is especially important because sphere formation has been reported to be an independent predictor of clinical outcome [45].

The number of spheres is assumed to be both an indicator of the aggressiveness of the tumor and the number of tumor initiating cells. The ability to form spheres strongly correlates with tumor growth and survival also in animal models [46]. Our results from intracranial transplantation into SCID mice were in agreement with this as mice transplanted with GICs featuring *NAT12/NAA30* gene knockdown survived significantly longer than controls. Altogether 3/11 mice did not form tumors until the end of the observation period in the *NAT12/NAA30* knockdown group while all 6/6 mice died from tumors in the control group.

There are few known substrates of NAT-C, but based on the Nt-amino acid composition of target proteins NAT-C can potentially Nt-acetylate up to 14.5 % of all cytoplasmic human proteins [47]. The N-terminus of MTOR protein begins with Met-Leu and is a strong candidate as a direct substrate [23, 24]. In a study in zebrafish TOR was suggested as a downstream target of NAT-C [24]. The authors also demonstrated that overexpression of TOR rescued the effect of depletion of a NAT-C subunit [24]. Knockdown of MTOR is a critical effector of the PIK3-AKT pathway which is dysregulated in many cancers [48]. In GBM AKT-signaling activity is significantly correlated with phosphorylation of MTOR [49].

In our study, knockdown of *NAT12/NAA30* led to decreased levels of phospho-MTOR (Ser2448), while the total MTOR levels remained unchanged (Fig. 5a). Phospho-MTOR (Ser2448) binds to the effector complexes mTORC1 and mTORC2 and is important for mTORC1 activity [50]. Implication of MTOR as a (direct or indirect) target of *NAT12/NAA30* was further supported by the expression analysis that showed increased levels of *IGFBP3*, an inhibitor of IGF1/MTOR pathway, in the cell cultures featuring *NAT12/NAA30* knockdown (Additional file 8: Figure S6).

Our results also indicate that *NAT12/NAA30* acts via the p53 pathway in GICs. In addition to an increment in p53 protein levels in the knockdown cultures we also provide evidence for dysregulation of the p53 pathway genes. Genes involved in cell-cycle regulation, apoptosis and DNA repair were those that suffered the most substantial expressional alteration (Additional file 8: Figure S6). We also show that *NAT12/NAA30* affects a considerable number of genes that regulate cell proliferation and protein kinases.

We investigated the expression of stem cell related genes in *NAT12/NAA30* knockdown cultures. Of the tested stem cell markers, we could detect reduced levels of nestin and SOX2 at the protein level. The knockdown of *NAT12/NAA30* also reduced the percentage of CD133+ cells. Our data thus indicate the effect of *NAT12/NAA30* knockdown on stemness in GICs.

A previous study has shown that mitochondrial proteins are substrates of NAT12/NAA30 [22]. Interestingly, we found that knockdown of *NAT12/NAA30* in GICs causes a more abrupt and severe mitochondrial membrane depolarization compared to the NS control cultures when these cells are exposed to hypoxia. This indicates a reduced mitochondrial tolerance to acute hypoxia upon *NAT12/NAA30* knockdown. This notion was further supported by microarray analysis showing that the role of *NAT12/NAA30* in hypoxia response involves *HIF1*α and several other genes (Additional file 10: Figure S9). Importantly, western blot analysis confirmed the reduced expression of HIF1α protein (Fig. 5d). Hypoxia induced factors are key mediators in the response of cancer stem cells to hypoxia [32, 51]. Several studies have pointed out that GICs reside in the hypoxic regions of GBMs [33] and that hypoxia-induced molecular changes regulate and maintain the phenotype of GICs [52]. This might imply that targeting *NAT12/NAA30* increases the vulnerability of GICs to hypoxic conditions.

## Conclusions

Although N-terminal acetylation complexes have lately received a lot of attention, the functional role of the NAT-C complex in cancer stem cells has not been investigated. In our work, we show that the knockdown of its catalytic subunit, *NAT12/NAA30,* reduced tumorigenic features of GICs and confirmed that MTOR and HIF1α are downstream targets of *NAT12/NAA30* in GICs. We therefore propose that, *NAT12/NAA30,* might serve as a potential therapeutic target in GBM. Our study further indicates involvement of *NAT12/NAA30* in gliogenesis and regulation of the p53 pathway. Specific inhibitors against NATs are under development [53] and might represent promising candidates for clinical testing.

## Materials and methods

### Tissue specimens and cell culture

Tissue specimens were harvested from consenting patients after approval by the Norwegian National Committee for Medical Research Ethics. Tumor biopsies were obtained as a part of surgical procedures for treating GBM. Normal brain tissue (SVZ, HPC, GM and WM) was obtained from fresh human temporal lobes surgically resected to treat medically refractory epilepsy. All biopsy specimens were evaluated by neuropathologists.

Tumor biopsies underwent mechanical dissociation and Trypsin-EDTA (Gibco, Life Technologies, NYC, NY, USA) was added for enzymatic dissociation. Subsequently, 2 mg/ml human albumin (Octapharma pharmazeutika produktionges, Vienna, Austria) was used to block the Trypsin effect and the cells were washed in L-15 (Lonza, Basel, Switzerland) before being plated in serum-free neurosphere medium containing 10 ng/ml basic fibroblast growth factor (bFGF) and 20 ng/ml epidermal growth factor (EGF) (both R&D Inc., Minneapolis, MN, USA), B27-supplement (1:50, Invitrogen, Carlsbad, CA, USA), 100 U/ml Penicillin/streptomycin (Lonza), 1 ng/ml Heparin (Leo Pharma, Ballerup, Denmark) and 8 mM Hepes (Lonza) in Dulbecco’s modified essential medium with nutrient mix F-12 and Glutamax (DMEM/F12, Invitrogen) [24, 25]. Dissociated GBM biopsies grown as free-floating spheres in serum free-medium containing mitogens (EGF and bFGF) are highly enriched for GICs [7, 54]. The GIC cultures used in this current work (T65, T08, T59) were analyzed for the expression of stem cell markers by flow cytometry and showed high expression of CD44, CXCR4, CD166 and CD9 while the expression of CD133 and CD15 was variable [55]. Immunolabeling showed that most cells were nestin and Sox2 positive (Additional file 6: Figure S4H-I, Stangeland, B. et al., in press). The cells were cultured in 75 cm^2^ non-treated flasks (Nunc, Roskilde, Denmark) at a density of 10^5^ cells/ml and supplemented with EGF and bFGF twice a week. When the spheres reached approximately 100 μm in diameter they were dissociated into single cells as previously described [28]. We have previously shown that the GIC cultures T65, T08, TC3 and TC4 formed invasive tumors upon orthotopic transplantation to SCID mice [55, 56].

NFCs (ReNcell VM Human Neural Progenitor Cell Line, SCC008, Merck Millipore, Darmstadt, Germany) were cultured as spheres in serum-free Neurobasal A medium (Gibco) containing B27 (Gibco), 2 mM L-glutamine, 10 ng/ml bFGF, and 20 ng/ml EGF (both from R&D Systems).

Tissue specimens from the adult human brain were dissociated into single cells and cultured according to our *FAILSAFE* protocol (1 % FBS, 10 ng/ml bFGF and 20 ng/ml TGF α) that ensures robust long-term propagation of multipotent stem cells from the adult human brain [57].

### Microarray analysis and public database mining

Microarray analysis was performed using Illumina gene chip. The data were quantile normalized and analyzed using J-Express (Molmine, Bergen, Norway) analysis software. We used published microarrays [57] with submission numbers GSE41470 (encompassing GSE41390, and GSE41394), GSE53800 (GSM1301030, GSM1301033, GSM1301042) as well as the microarrays submitted in connection with this work GSE60818 (encompassing GSE60705 and GSE60706). For more information on microarray analysis see Additional file 1: Figure S1 and Additional file 2: Figure S2 and Additional file 3 and Additional file 7.

REMBRANDT: Microarray data from the Repository for Molecular Brain Neoplasia Data (REMBRANDT, National Cancer Institute, 2005, http://rembrandt.nci.nih.gov) were accessed on January the 15th 2014. Hierarchical clustering with distance matrix and the K-means clustering of the NAT genes’ expression were performed using J-Express software (Molmine). For calculation of statistical parameters, we used Graphpad Prism (www.graphpad.com).

### Western blot and quantification of protein expression

The cells were homogenized by triturating in Cell Extraction Buffer (Mammalian cell extraction kit, Biovision, Milpitas, CA, USA) and centrifuged through a QIAshredder (Qiagen, Venlo, Netherlands). For HIF1α and the western blots with phospho-proteins, the cells were homogenized in 10 mmol/L Tris–HCl (pH 7.4), 1 % SDS, 10 mmol/L NaF, and 2 mmol/L Na3VO4 as previously described [58]. 20–40 μg of whole protein extract were mixed with loading buffer (NuPAGE, Invitrogen) and loaded onto a 4–12 % gradient Nu-PAGE gel (Invitrogen). Protein gels were blotted onto 0.45 μm PVDF membranes (Invitrogen). Membranes were blocked with 5 % skimmed milk in TBS/0.1 % Tween 20 (TBST) and probed with primary antibodies diluted in the same solution. Primary antibodies from Cell Signaling Technologies (CST, Danvers, MA, USA) were incubated in bovine serum albumin (BSA) according to recommended procedures. For a complete list of the antibodies used in this study see Additional file 12. For a complete list of the antibodies used in this study see Additional file 12. The blots were developed using the Lumiglo Reserve CL Substrate kit, and detected using the Epi Chemi II Darkroom (UVP-Laboratory Products, Upland, CA, USA). The intensities of the protein bands were quantified using Photoshop (Adobe, San Jose, CA, USA), background corrected and normalized to the intensities of the corresponding ACTB bands, so that the relative protein expression (RPE) could be calculated (RPE_protx_ = PE_protx_/PE_ACT_).

### RNA isolation and real-time quantitative reverse-transcription PCR (qPCR)

Total RNA was isolated using an RNeasy Mini Kit and QIAshredder (Qiagen). For cDNA synthesis, experimental set up and oligonucleotide design we used the procedure previously described [59]. For expression analysis of *NAT12/NAA30* we used seven sets of oligonucleotides covering translated and non-translated regions of the reference sequence NM_001011713.2. For oligonucleotide sequence information see Additional file 3. qPCR was performed on an ABI PRISM 7900HT PCR machine (Applied Biosystems, Life Technologies, Foster City, Ca, USA) using SYBR Premix Ex Taq™ (Takara, Otsu, Japan) or Taqman probes (Applied Biosystems) according to the manufacturer's protocol. For analysis of the *NAT12/NAA30* coding regions we used the following Taqman probes: ex2-3 (Hs02340852), ex3-4(Hs02340853) and ex4-5(Hs02340854). Crossing point (CP) values were generated using second-derivative calculation software (SDS2.2). Gene expression levels in GIC cultures were calculated using two house-keeping genes and multiple controls (values obtained for all tested NSCs and NFCs). For analysis of qPCR results we used 2-∆∆ CT-method and REST software [60].

### RNAi-mediated gene knockdown

GIC culture T65 was used to establish stable *NAT12/NAA30* knockdown cultures using shRNA1 (RHS4430-99149700; Clone ID V2LHS_180063), shRNA2 (RHS4430-99137454; Clone ID V2LHS_180058) and a non-silencing (NS) shRNA as a control (RHS4346) (all from Open Biosystems, Thermo Scientific, Huntsville, AL, USA). Cell cultures harboring shRNA constructs 1 and 2 are referred to as KD1 and KD2 respectively.

Production of virus in the 293 FT cell line using plasmid DNA and concentration of virus was done as following: Nine μg of plasmid DNA was transfected into the 293FT cell line using Arrest-In transfection reagent (according to the manufacturer’s protocol). Viral supernatants were collected after 48 and 72 h, centrifuged at 3000 rpm for 20 min at 4 °C and filtered through a sterile 0.45 μm low protein binding filter (Sarstedt, Nümbrecht, Germany). The virus was then concentrated in sterile SW28 ultracentrifuge tubes by ultracentrifugation (Beckman Optima™ LE-80 K ultracentrifuge, Fullerton, CA, USA) equipped with a SW-28 rotor at 23,000 rpm for 1.5 h at 4 °C. The pellet was resuspended in 200 μl of DMEM and aliquots of the concentrated virus were stored at −80 °C. GSC cultures (10×10^4^ cells/ well) were then transduced in 24-well plates by adding 10 μl concentrated virus/well and the cells were incubated for 48 h at 37 °C in 5 % CO_2_. Three to five days after transduction, GFP positive cells were selected by FACS sorting using a FACS Diva cell sorter (Becton Dickinson, Franklin Lakes, NJ, USA) equipped with an argon ion laser, ‘TurboSort Plus’ option, and Diva software (Becton Dickinson). Alternatively, the cells were selected in 2 μg/ml Puromycin (Sigma-Aldrich, St. Louis, MO, USA) for 3–4 weeks before use for functional assays.

### Cell viability, apoptosis and sphere-forming assay

For analysis of cell viability we used a colorimetric test based on tetrazolium salt – XTT (Roche Diagnostics, Indianapolis, IN, USA). Briefly, after sphere dissociation single cells were plated in neurosphere medium at a density of 1×10^4^ cells per well in a flat-bottom 96-well plate (Sarstedt). Cells were incubated overnight and XTT-reagents added as recommended by the manufacturer. The colorimetric changes (absorbance) were measured after 24 h at 490 nm using a plate reader (Victor™ *X*5 Multilabel Plate Reader, Perkin Elmer 2030, Waltham, MA, USA). Five wells were evaluated for each cell culture. All results were presented as a mean of three independent experiments ± standard deviation. P-values were calculated using unpaired *t*-test with Welch’s correction.

Apoptosis was assessed using a fluorimetric assay which measures the activity of activated caspases (Roche Diagnostics). 1×10^4^ cells were plated per well in a flat-bottom 96-well plate (Sarstedt) and incubated overnight before adding the reagents as recommended by the manufacturer. The fluorimetric changes were measured after 24 h at 485/535 nm using a plate reader (Victor™ *X*5 Multilabel Plate Reader). Five wells were evaluated for each cell culture.

The sphere-forming assay was done by seeding single cell suspensions containing 500 cells per well in ultra-low attachment flat bottom 96-well plates for 10 days (Sarstedt). Subsequently, the plates were imaged using GelCount™ (Oxford Optronics, Abington, UK). Only spheres >50 μm were taken into consideration and 10 wells were evaluated for each cell culture. The number and the size (average area) of spheres were measured using software supplied by the manufacturers. All results were presented as a mean of five independent experiments ± standard deviation. P-values were calculated using unpaired *t*-test with Welch’s correction.

### Intracranial transplantation to SCID mice

All animal procedures were approved by the National Animal Research Authority. C.B.-17 SCID male mice (8–9 weeks old) were obtained from Taconic (Ejby, Denmark). They were acclimatized for > 1 week and divided into 3 groups: (i) T65 NS cells (*n* = 6), (ii) T65 KD1 cells (*n* = 6) and (iii) T65 KD2 cells (*n* = 6).

T65 GIC cultures were sorted by FACS after viral transduction and cultured for a few passages before transplantation. After tumor sphere dissociation, cells were counted and plated at a low density for overnight incubation. Prior to the inoculation of cells, mice were anesthetized and placed in a stereotactic frame (Kopf Instruments, Tujunga, CA, USA) and ~3×10^5^ incubated cells were inoculated into the right striatum (AP 0 mm, RV 2 mm, ML 2 mm) using a Hamilton syringe and needle (Hamilton, Bonaduz, Switzerland) [28].

All animals were monitored for adverse effects and examined regularly for general appearance, signs of distress, and weight measurements taken. Mice were sacrificed when they lost >10 % of their bodyweight and developed neurological symptoms. Brains were harvested and further processed for HE-staining as described previously [28]. Results for all groups are presented as median survival in days. Log-rank test was used to calculate the level of significance.

### Mitochondrial depolarization assay

After passage, KD1, KD2 and NS cultures were seeded on fibronection-coated cover slips, and cultured in neurosphere medium in separate culture dishes for 1–6 days before experiments.

For each experiment, a cover slip was removed from its cell culture dish, and mounted in the microscopy chamber. Artificial CSF (aCSF) with the fluorescent cationic dye Rhodamine 123 (30 μM) (Invitrogen) was added to the chamber, and cells were loaded with dye for 15 min at 37 °C on the microscope. The chamber was covered with a lid, and subsequently perfused with air and 5 % CO_2_ for maintenance of appropriate pH. After loading of dye, the chamber was perfused with aCSF at a flow rate of approximately 1 ml/min. Imaging was started after at least 10 min of flow. Healthy looking cells were chosen by the criteria clear cell membrane and intact processes. Live imaging was performed using an Olympus IX81 inverted microscope equipped with a MT20 fluorescence light unit and Olympus *xcellance* software. Images were captured every 15 s. Rhodamine 123 (Rh123) used at 30 μM concentration accumulates in healthy mitochondria and is released to the cytosol upon depolarization of the mitochondria [61]. This release is shown by increment in fluorescence from the cell. Since all cells expressed GFP, the red spectrum of Rh123 was recorded by using filters suitable for red fluorescent protein.

The cells were exposed to severe hypoxia for 15 min. Hypoxia was obtained by removing most of the oxygen by nitrogen perfusion of the flow solution. The oxygen scavenger Sodium Dithionite (0.75 mM) (Sigma-Aldrich) induced severe hypoxia. Control experiments were done with Sodium Dithionite perfusing the fluid with oxygen to control for any toxic effects other than caused by hypoxia. These control experiments did not show toxic effects on mitochondrial function. The protonophore Carbonyl cyanide 4-(trifluoromethoxy) phenylhydrazone (FCCP) (1 μM) (Sigma-Aldrich) was applied before the end of all experiments. FCCP fully depolarizes mitochondria, and thereby causes release of any remaining dye from the mitochondria. In this way any remaining mitochondrial membrane potential in the cell is revealed by an increase in Rh123 fluorescence.

### Immunolabeling

GIC cultures were plated in 24-well plates (Sarstedt) or chamber slides (Sigma-Aldrich) pretreated with Retronectin 50 μg/ml (Takara) and incubated overnight to facilitate cell adhesion. Cells were fixed with 4 % PFA and washed with PBS. Tissue slides were fixed in 10 % buffered formalin and washed with PBS. Immunolabeling was performed as previously described [62]. Briefly, permeabilization in 0.1 % Triton, was followed by blocking with 5 % BSA and 5 % blocking serum, and incubations firstly with primary antibody in 0.1 % Tween-20 (o/n) and then with secondary antibody (2 h). The protocol was slightly modified for the co-staining of NAT12/NAA30 and CD31 and 0.1 % Triton was replaced with 0.1 % (w/v) Saponin (Sigma-Aldrich). After staining of nuclei with Hoechst 33342 (Invitrogen) the slides were mounted with antifade reagent (ProLong Gold, Invitrogen).

For detection of NAT12/NAA30 protein we used the primary antibody #AV48508 [(Sigma-Aldrich), rabbit 1:200]. For confirmation of the obtained results we used #NBP1-70631 [(Novus Biologicals, Littleton, CO, USA), rabbit 1:200]. Immunofluorescence images shown in the article were obtained with #AV48508. We used primary antibodies against human nestin (NES) [#ab6320 (Abcam, Cambridge, UK), mouse 1:400], CD31 [#ab54211 (Abcam), mouse 1:100] and SOX2 [#AF2018 (R&D), goat 1:100].

Secondary antibodies were: Alexa Fluor 488 [#A-11059 (Invitrogen), donkey anti-mouse, 1:500], Alexa Fluor 555 [#A-31572 (Invitrogen), donkey anti-rabbit, 1:500], Alexa Fluor 594 [#R37119 (Invitrogen), donkey anti-rabbit, 1:500], and Alexa Fluor 594 [#A-11058 (Invitrogen), donkey anti-goat, 1:500].

### Flow cytometry

Spheres from GIC culture T65 were dissociated into single cells, and incubated overnight to recover. Cells were pelleted, and incubated with primary conjugated antibodies against CD133/2 –PE conjugated (Miltenyi biotec) and SSEA1-PE (Becton-Dickinson). Cells were then analyzed using a LSRII flow cytometer (Becton-Dickinson).

## Additional files

Additional file 1: Figure S1. Microarray analysis of the selected NAT genes. **A**, Expression of *NAA30/NAT12* in tissues and cells. Expression was measured by microarray analysis of 17 cell cultures (NSCs, NFCs and GICs) and four tissue specimens from normal and GBM tissues. Median expression in cell cultures was 126.8 while in tissues it was 94.29 with p value of 0.0013. **B**-**H**, Expression of the selected NATs in NSCs, GICs, normal brain tissues, GBM and NFCs. P values were calculated using the Mann Whitney test. Due to small sample sizes the p values proved reliable only for GICs vs. NSCs and were as follows: p(NAT12)=0.023, p(NAT6)=0.0002, p(NAT14)=0.78, p(NAT13)=0.3231, p(NAT8L)=0.0057, p(NAT8)=0.0006, p(NAT8B)=0.003 and p(NAT10)=0.4331. Additional file 2: Figure S2. K-means clustering of the expression data for the selected NAT genes. Additional file 3: Supplementary File 1. Oligonucleotides and microarray probes. (PDF 115 kb) Additional file 4: Supplemetary File 2. Additional qPCR statistics for Fig. 1, Fig. 3 and Fig. 5. (PDF 254 kb) Additional file 5: Figure S3. Kaplan-Meier survival plot for NAT genes (*NAT12*, *NAT1*, *NAT2* and *NAT10*). P values (Log-rank test) signify the differences in patient survival in the selected groups with the following parameters: Up-Regulated: fold ≥ 2; Down-Regulated: fold ≥ 2. Reporter Type: Affymetrix. Additional file 6: Figure S4. Protein expression of NAT12/NAA30 detected with an anti-NAT12/NAA30 antibody (red) in biopsy specimens from normal brain (A), in GBM (B), in NSC (C-D) and GIC cultures (E-F). Cryostat sections with HE-staining from xenograft experiments showed that the control cultures (T65 and T65 NS) form visible tumors upon intracranial transplantation in contrast to the *NAT12/NAA30* knockdowns (KD1 and KD2) (G). High magnification images showed that tumor cells are present in all groups even though the tumors are not visible in the knockdowns. Protein expression of NESTIN in GIC culture T08 (H) and SOX2 in T65 (I). Additional file 7: Supplementary File 3. Functional annotation based on the microarray analysis of *NAT12/NAA30* knockdown cultures. (XLSX 201 kb) Additional file 8: Figure S5-S7.
**Figure S5.** *NAT12/NAA30* knockdown resulted in dysregulation of ribosome assembly as shown by microarray analysis. Using the DAVID functional annotation tool we analyzed the KEGG pathway “Ribosome” where 13 genes (p=5.6E-06) were differentially regulated in KD1 and KD2. See also Supplementary File 3. Dysregulated genes are marked with red asterisks. **Figure S6.** *NAT12/NAA30* knockdown resulted in dysregulation of the p53 pathway as shown by microarray analysis. Using the DAVID functional annotation tool we analyzed the KEGG pathway “p53” where 8 genes (p=3.1E-03) were differentially regulated in KD1 and KD2. See also Supplementary File 3. Dysregulated genes are marked with red asterisks. **Figure S7.** *NAT12/NAA30* knockdown resulted in dysregulation of sphingolipid metabolism as shown by microarray analysis. Using the DAVID functional annotation tool we analyzed the KEGG pathway “Sphingolipid metabolism” where 6 genes (p=4.8E-03) were differentially regulated in KD1 and KD2. See also Supplementary File 3. Additional file 9: Figure S8. Expression of the selected genes and proteins in the NAT12/NAA30 knockdown cultures. **A**, Western blot showing that the protein levels of active β-Catenin (ABC), phospho-p70 S6 kinase (Thr 389), phospho-STAT3 (Ser727) and GLI1 were slightly affected or not altered in the knockdown cultures. The signals obtained with Anti-SHH and Anti-phospho-p53 (Ser15) were below the detection level of western blot (not shown). For a complete list of ABs see Supplementary File 4. **B**, Western blots showing the expression of neural stem cell markers SOX2 and NESTIN, proteins representing hypoxia response (KCNMA1), cell cycle (CCND1) and ACTB (ACTIN). **C**, Western blots showing the expression of p-AKT (Ser473), total AKT, p-AKT (Ser308), p-p4E-BP1 and total p4E-BP1. **D**, Expression of selected stem cell genes SOX2, NANOG, POU5F1/OCT4, MYC and CCND1 on microarrays (n=4 for each cell culture). None of the selected genes were differentially regulated in the NAT12/NAA30 knockdown cultures. The bottom and top of each box indicate the 25th and 75th percentile (the lower and upper quartiles, respectively), and the band near the middle of the box represents the 50th percentile (the median). Interestingly, the knockdown strength was in correlation with the effect on downstream genes. In the KD2 culture that exhibited stronger knockdown effect at the protein level and better reduction in cell viability and number of spheres we could also detect decreased levels of GLI1, nestin and phospho proteins such as phospho-AKT (Ser308) and p-4E-BP1(Thr37/46) (Figure S8 A-C). Additional file 10: Figure S9.
*NAT12/NAA30* knockdown resulted in dysregulation of the hypoxia response pathway as shown by microarray analysis. Asterisks signify p values and indicate level of significance. *=(p≈0.01-0.05), **=(p≈0.001-0.01). Additional file 11: Figure S10.
*NAT12/NAA30* knockdown resulted in reduction of CD133+ cells as shown by flow cytometry. Additional file 12: Supplementary File 4. List of antibodies used for western blot. (DOCX 53 kb)
