# Supplementary figures and images for: Knockdown of NAT12/NAA30 reduces tumorigenic features of glioblastoma-initiating cells
Source: Mol Cancer. 2015 Aug 21;14:160. doi: 10.1186/s12943-015-0432-z (PMC4546247; doi:10.1186/s12943-015-0432-z)

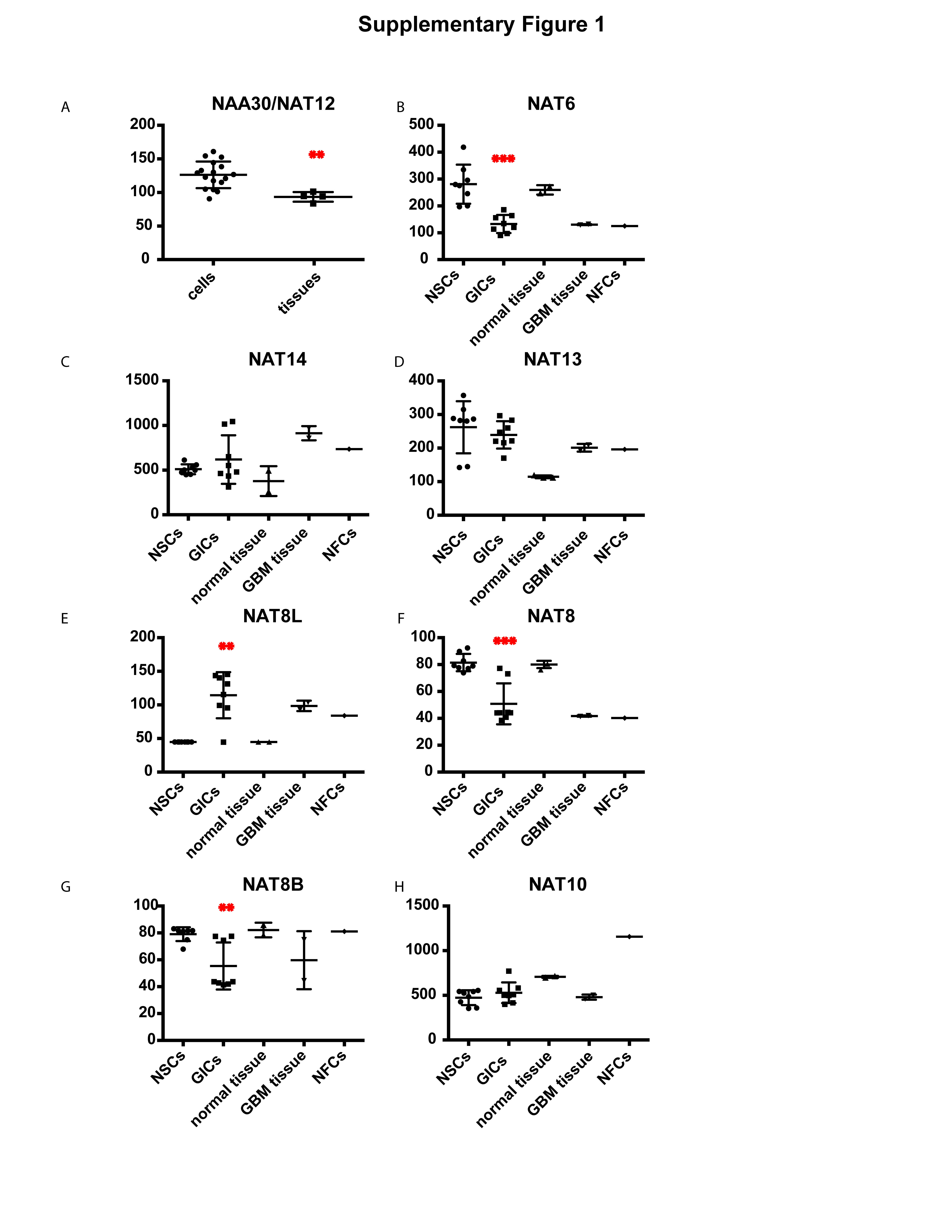

Supplement: Additional file 1: Figure S1. — Microarray analysis of the selected NAT genes. A, Expression of NAA30/NAT12 in tissues and cells. Expression was measured by microarray analysis of 17 cell cultures (NSCs, NFCs and GICs) and four tissue specimens from normal and GBM tissues. Median expression in cell cultures was 126.8 while in tissues it was 94.29 with p value of 0.0013. B-H, Expression of the selected NATs in NSCs, GICs, normal brain tissues, GBM and NFCs. P values were calculated using the Mann Whitney test. Due to small sample sizes the p values proved reliable only for GICs vs. NSCs and were as follows: p(NAT12)=0.023, p(NAT6)=0.0002, p(NAT14)=0.78, p(NAT13)=0.3231, p(NAT8L)=0.0057, p(NAT8)=0.0006, p(NAT8B)=0.003 and p(NAT10)=0.4331. [file 12943_2015_432_MOESM1_ESM.tiff]

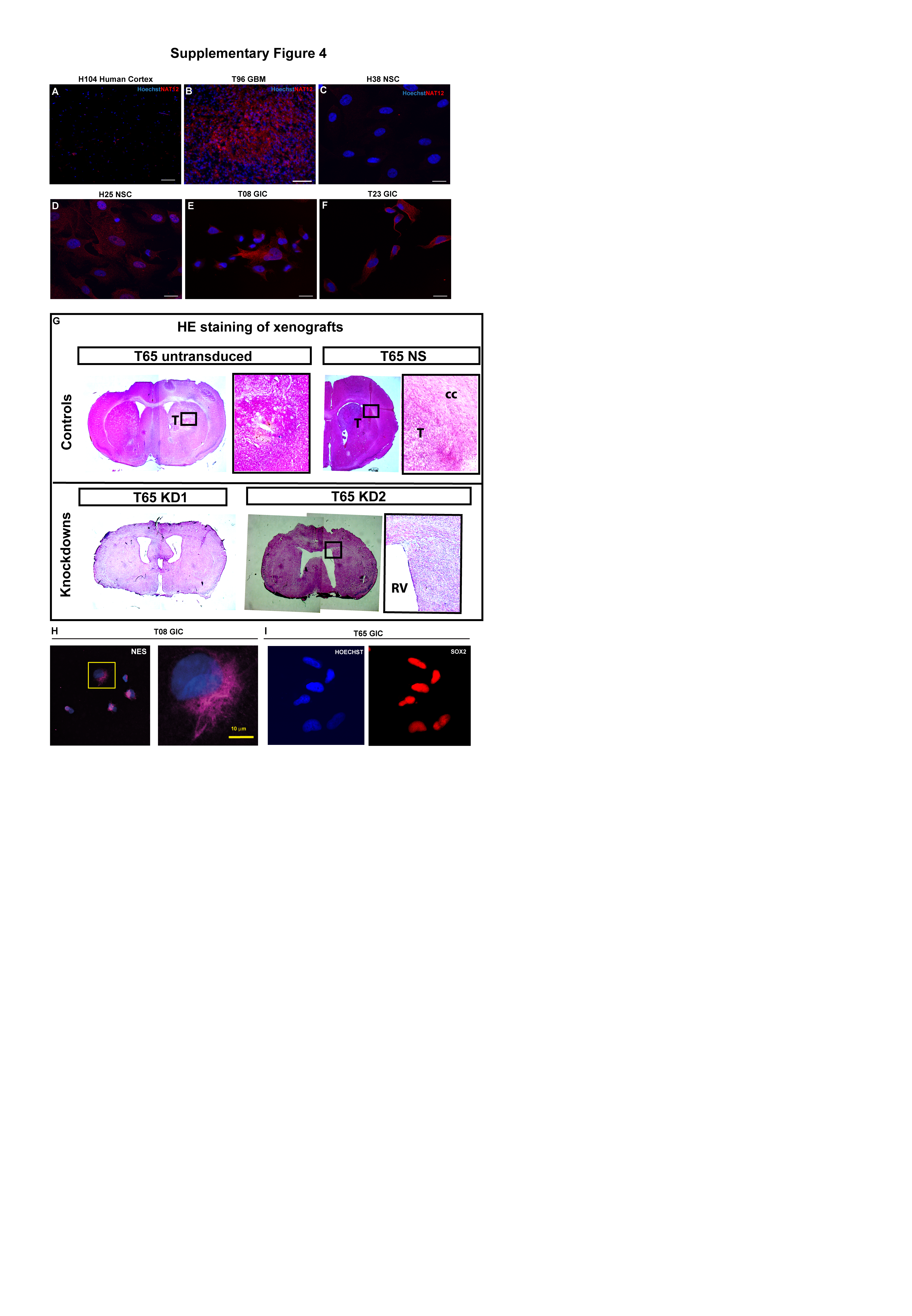

Supplement: Additional file 6: Figure S4. — Protein expression of NAT12/NAA30 detected with an anti-NAT12/NAA30 antibody (red) in biopsy specimens from normal brain (A), in GBM (B), in NSC (C-D) and GIC cultures (E-F). Cryostat sections with HE-staining from xenograft experiments showed that the control cultures (T65 and T65 NS) form visible tumors upon intracranial transplantation in contrast to the NAT12/NAA30 knockdowns (KD1 and KD2) (G). High magnification images showed that tumor cells are present in all groups even though the tumors are not visible in the knockdowns. Protein expression of NESTIN in GIC culture T08 (H) and SOX2 in T65 (I). [file 12943_2015_432_MOESM6_ESM.tiff]

## Supplementary Figure 6

### P53 SIGNALING PATHWAY

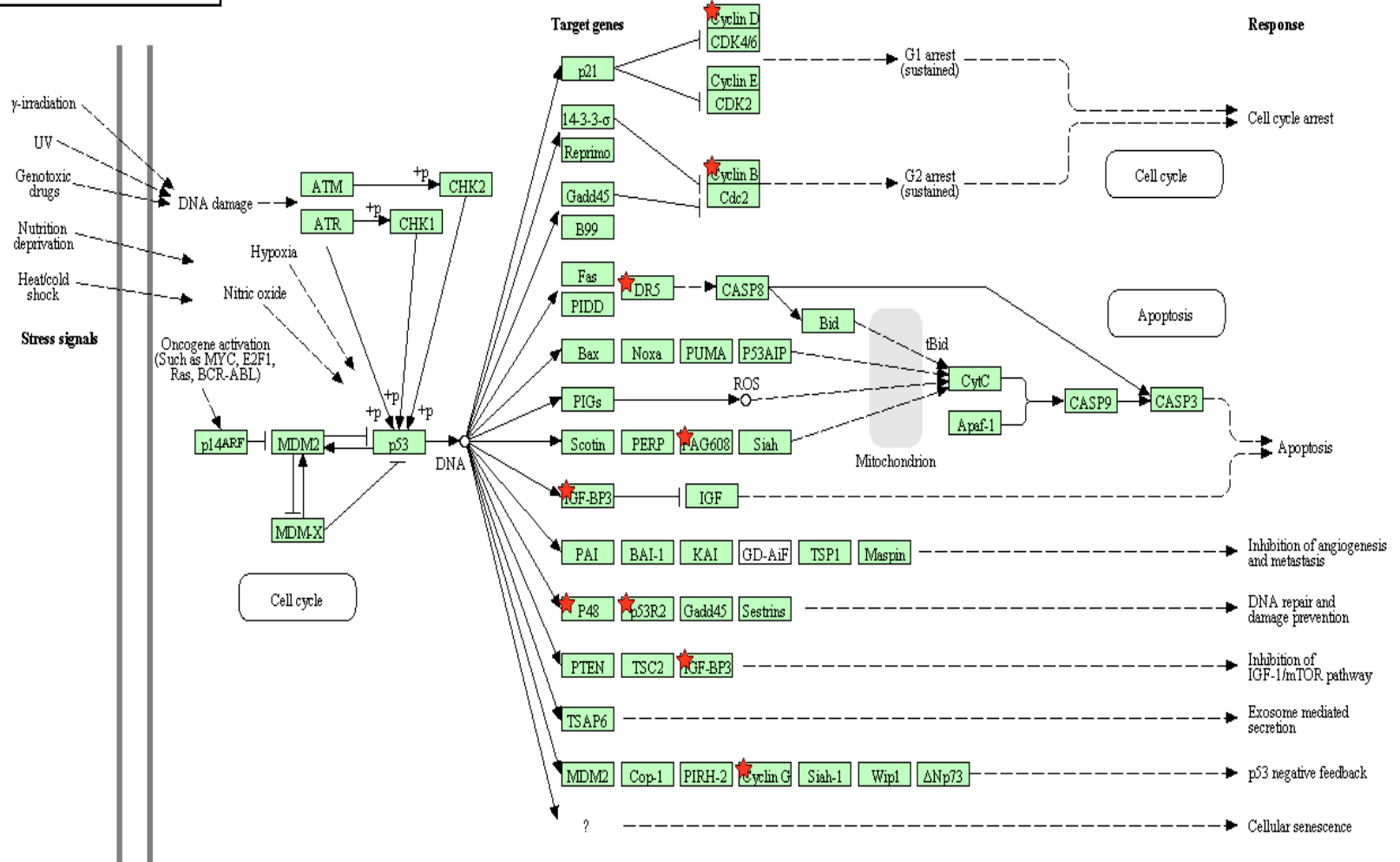

Supplement: Additional file 8: Figure S5-S7. — Figure S5. NAT12/NAA30 knockdown resulted in dysregulation of ribosome assembly as shown by microarray analysis. Using the DAVID functional annotation tool we analyzed the KEGG pathway “Ribosome” where 13 genes (p=5.6E-06) were differentially regulated in KD1 and KD2. See also Supplementary File 3. Dysregulated genes are marked with red asterisks. Figure S6. NAT12/NAA30 knockdown resulted in dysregulation of the p53 pathway as shown by microarray analysis. Using the DAVID functional annotation tool we analyzed the KEGG pathway “p53” where 8 genes (p=3.1E-03) were differentially regulated in KD1 and KD2. See also Supplementary File 3. Dysregulated genes are marked with red asterisks. Figure S7. NAT12/NAA30 knockdown resulted in dysregulation of sphingolipid metabolism as shown by microarray analysis. Using the DAVID functional annotation tool we analyzed the KEGG pathway “Sphingolipid metabolism” where 6 genes (p=4.8E-03) were differentially regulated in KD1 and KD2. See also Supplementary File 3. [file 12943_2015_432_MOESM8_ESM.zip › add8/1018244584147474_add5.pdf]

## Supplementary Figure 7

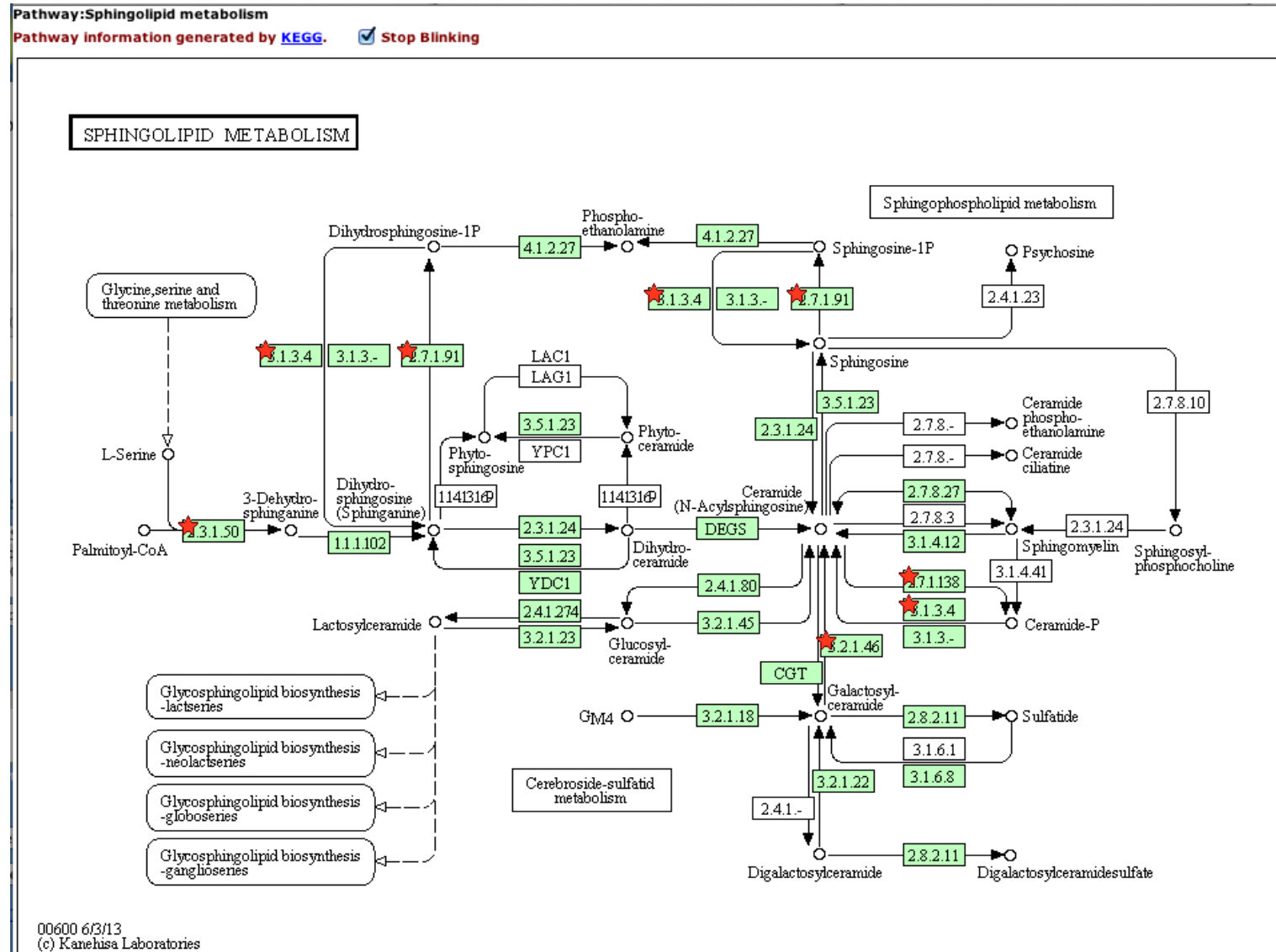

Supplement: Additional file 8: Figure S5-S7. — Figure S5. NAT12/NAA30 knockdown resulted in dysregulation of ribosome assembly as shown by microarray analysis. Using the DAVID functional annotation tool we analyzed the KEGG pathway “Ribosome” where 13 genes (p=5.6E-06) were differentially regulated in KD1 and KD2. See also Supplementary File 3. Dysregulated genes are marked with red asterisks. Figure S6. NAT12/NAA30 knockdown resulted in dysregulation of the p53 pathway as shown by microarray analysis. Using the DAVID functional annotation tool we analyzed the KEGG pathway “p53” where 8 genes (p=3.1E-03) were differentially regulated in KD1 and KD2. See also Supplementary File 3. Dysregulated genes are marked with red asterisks. Figure S7. NAT12/NAA30 knockdown resulted in dysregulation of sphingolipid metabolism as shown by microarray analysis. Using the DAVID functional annotation tool we analyzed the KEGG pathway “Sphingolipid metabolism” where 6 genes (p=4.8E-03) were differentially regulated in KD1 and KD2. See also Supplementary File 3. [file 12943_2015_432_MOESM8_ESM.zip › add8/1018244584147474_add6.pdf]

# Supplementary Figure 8

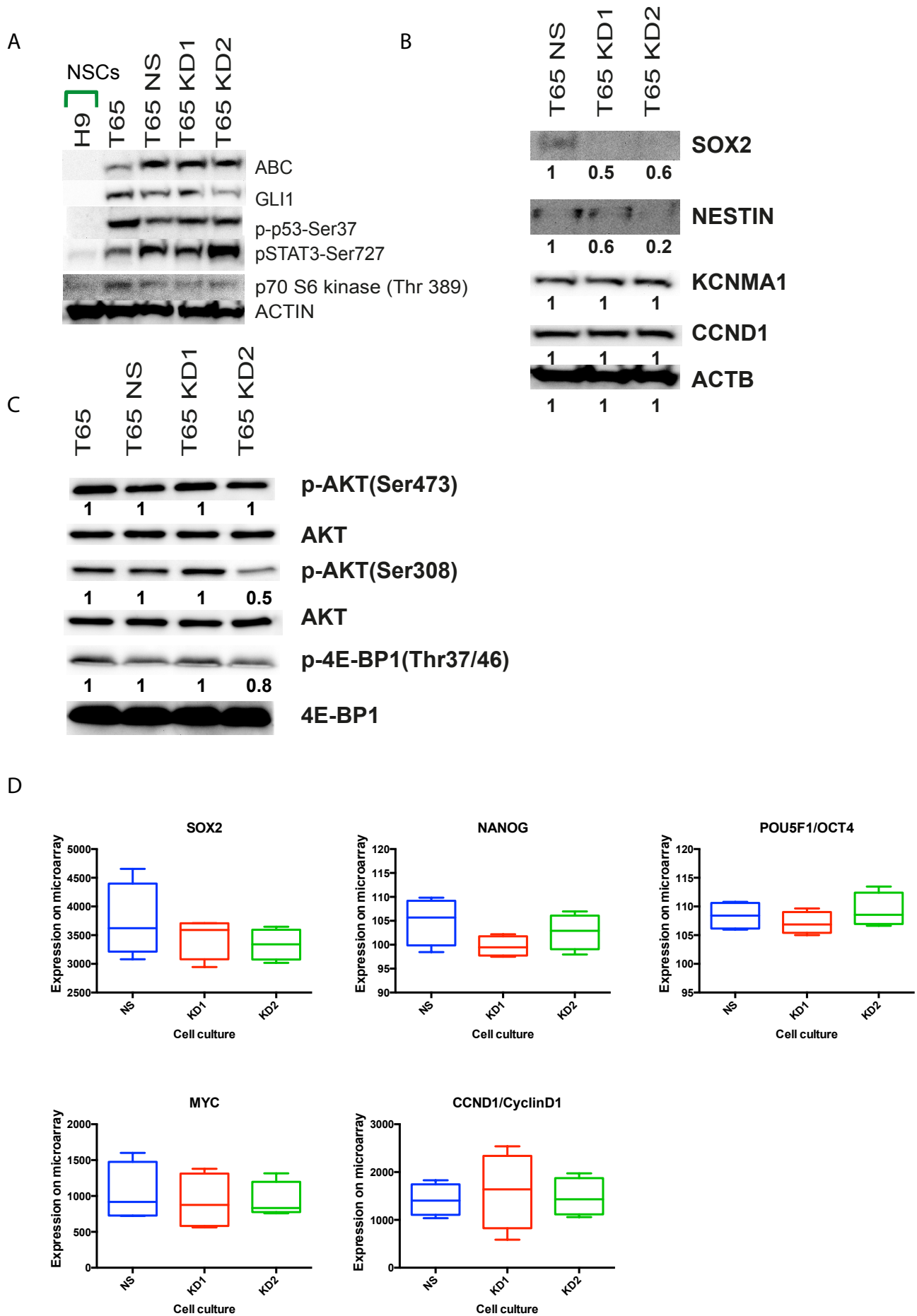

Supplement: Additional file 9: Figure S8. — Expression of the selected genes and proteins in the NAT12/NAA30 knockdown cultures. A, Western blot showing that the protein levels of active β-Catenin (ABC), phospho-p70 S6 kinase (Thr 389), phospho-STAT3 (Ser727) and GLI1 were slightly affected or not altered in the knockdown cultures. The signals obtained with Anti-SHH and Anti-phospho-p53 (Ser15) were below the detection level of western blot (not shown). For a complete list of ABs see Supplementary File 4. B, Western blots showing the expression of neural stem cell markers SOX2 and NESTIN, proteins representing hypoxia response (KCNMA1), cell cycle (CCND1) and ACTB (ACTIN). C, Western blots showing the expression of p-AKT (Ser473), total AKT, p-AKT (Ser308), p-p4E-BP1 and total p4E-BP1. D, Expression of selected stem cell genes SOX2, NANOG, POU5F1/OCT4, MYC and CCND1 on microarrays (n=4 for each cell culture). None of the selected genes were differentially regulated in the NAT12/NAA30 knockdown cultures. The bottom and top of each box indicate the 25th and 75th percentile (the lower and upper quartiles, respectively), and the band near the middle of the box represents the 50th percentile (the median). Interestingly, the knockdown strength was in correlation with the effect on downstream genes. In the KD2 culture that exhibited stronger knockdown effect at the protein level and better reduction in cell viability and number of spheres we could also detect decreased levels of GLI1, nestin and phospho proteins such as phospho-AKT (Ser308) and p-4E-BP1(Thr37/46) (Figure S8 A-C). [file 12943_2015_432_MOESM9_ESM.pdf]

# Supplementary Figure 9

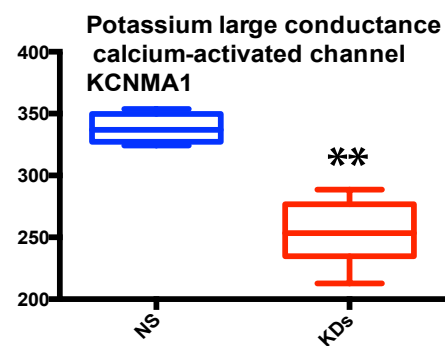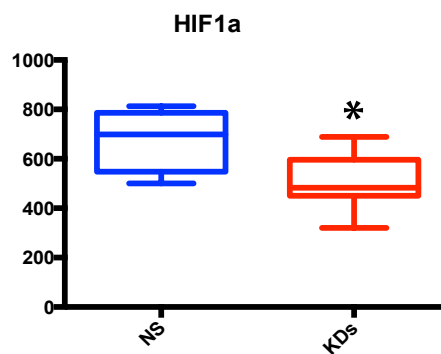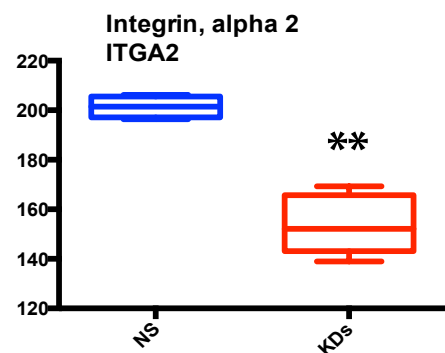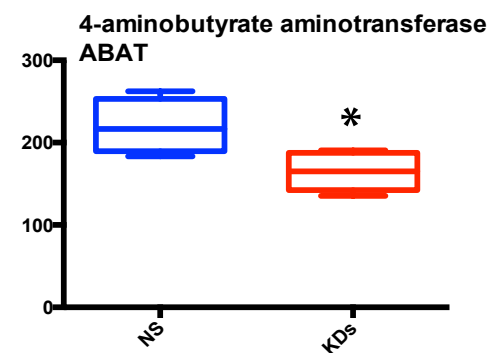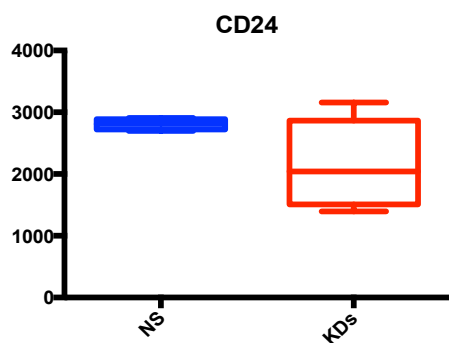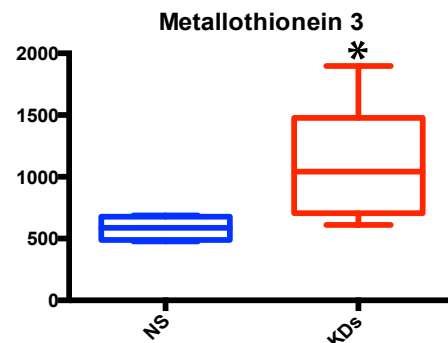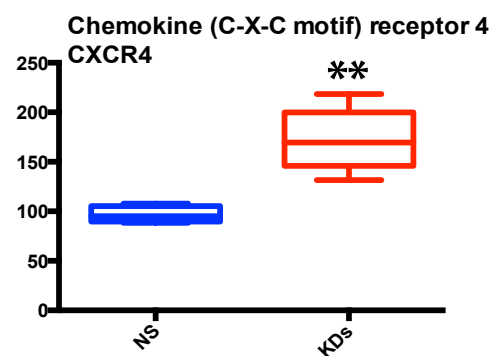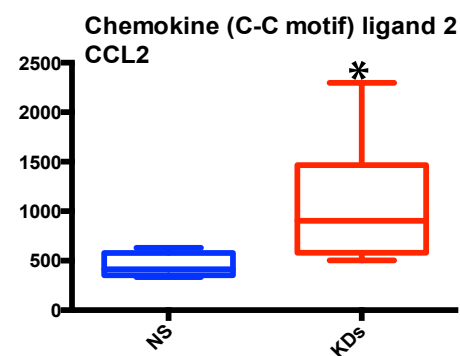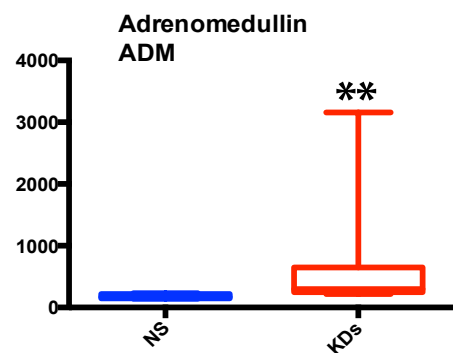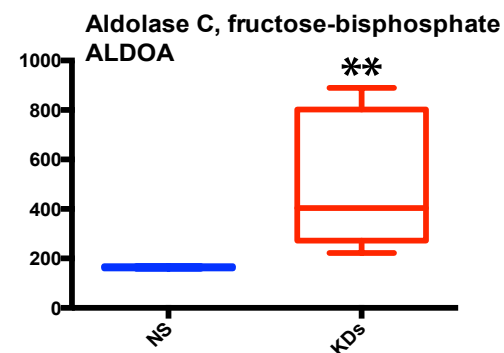

Supplement: Additional file 10: Figure S9. — NAT12/NAA30 knockdown resulted in dysregulation of the hypoxia response pathway as shown by microarray analysis. Asterisks signify p values and indicate level of significance. *=(p≈0.01-0.05), **=(p≈0.001-0.01). [file 12943_2015_432_MOESM10_ESM.pdf]

Supplementary Figure 10

NS-cntr

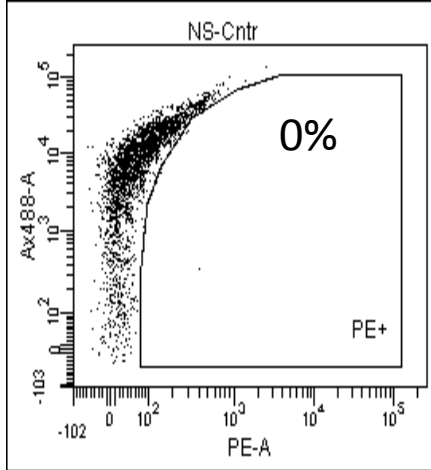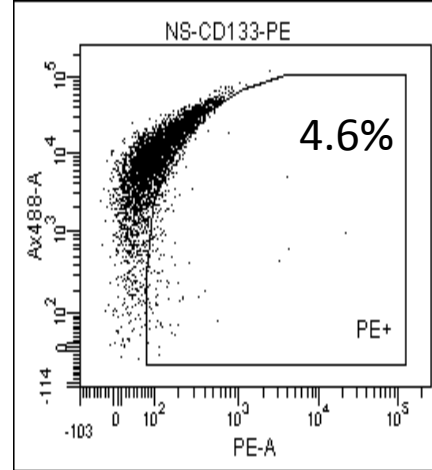

KD1

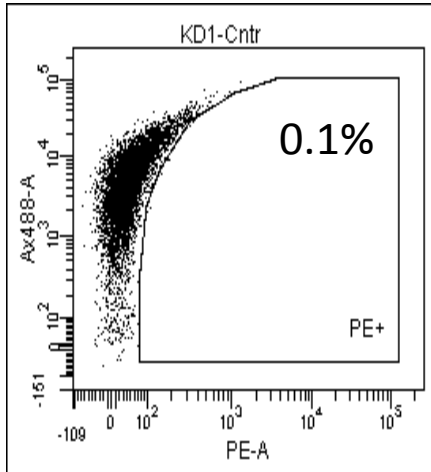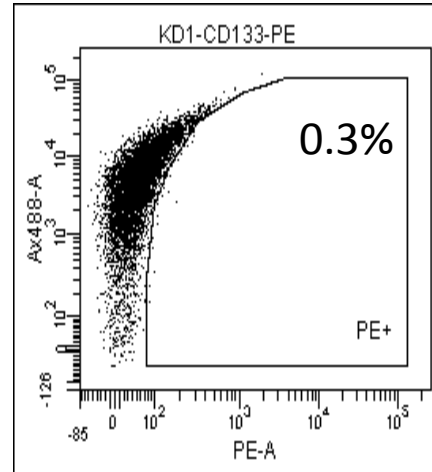

KD2

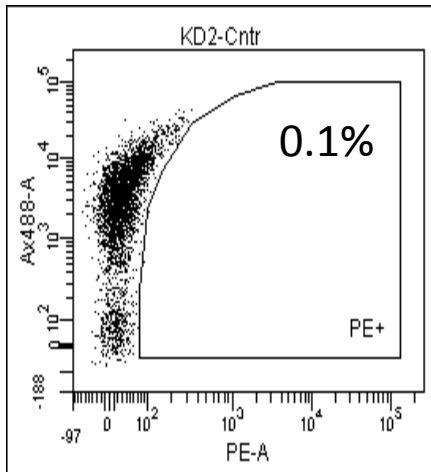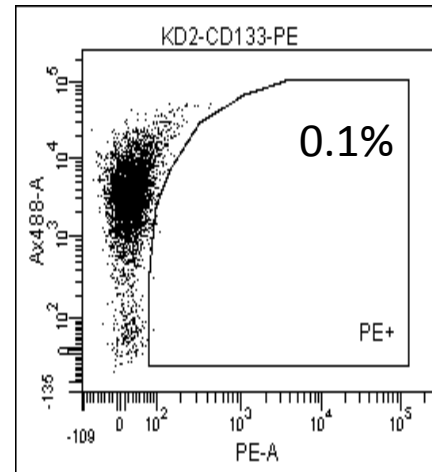

Supplement: Additional file 11: Figure S10. — NAT12/NAA30 knockdown resulted in reduction of CD133+ cells as shown by flow cytometry. [file 12943_2015_432_MOESM11_ESM.pdf]
