# Supplementary material for: Knockdown of NAT12/NAA30 reduces tumorigenic features of glioblastoma-initiating cells
Source: Mol Cancer. 2015 Aug 21;14:160. doi: 10.1186/s12943-015-0432-z (PMC4546247; doi:10.1186/s12943-015-0432-z)

Supplementary Figure 2

K-means clustring (Pearson 4 groups)

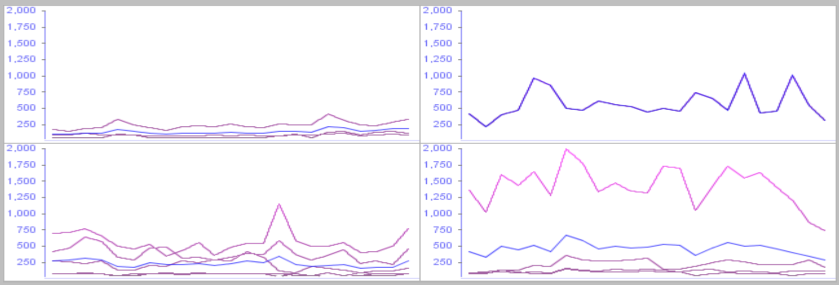

2	NAT11
6	NAT15
12	NAT8L

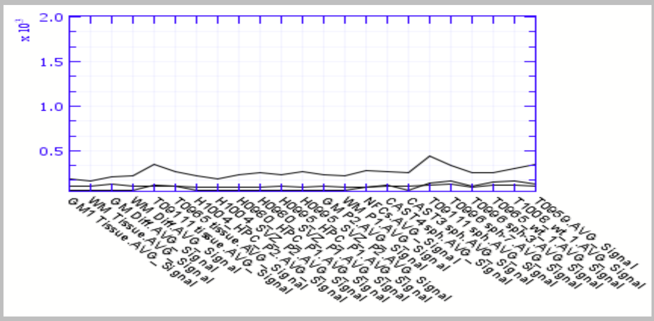

1	NAT10
7	NAT2
9	NAT6
10	NAT8
11	NAT8B
13	NAT9

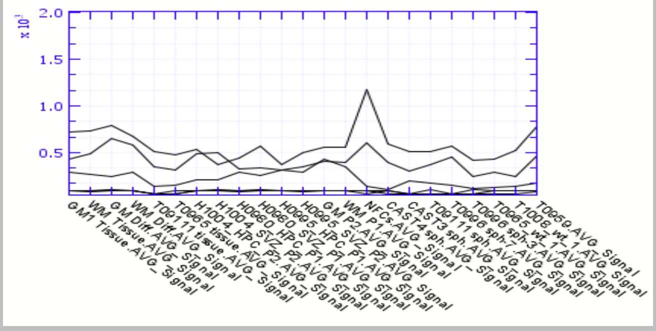

5	NAT14

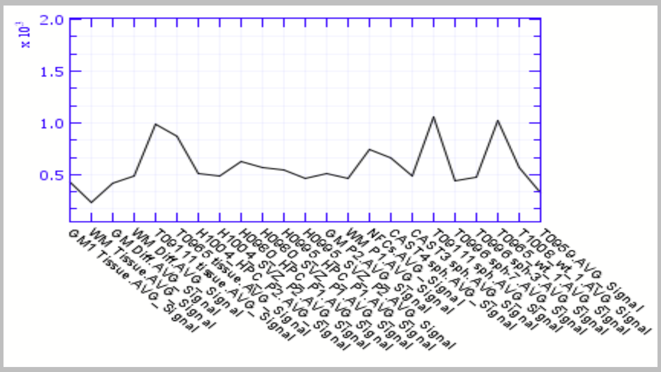

0	NAT1
3	NAT12
4	NAT13
8	NAT5

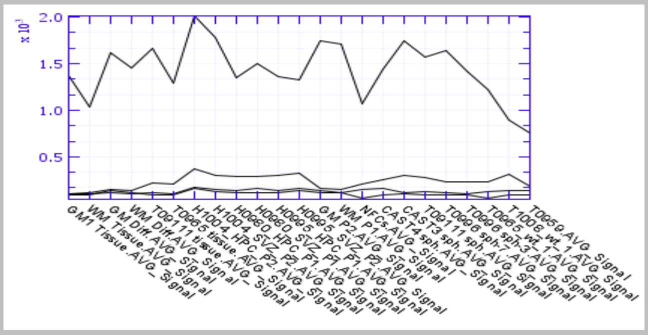

Supplement: Additional file 2: Figure S2. — K-means clustering of the expression data for the selected NAT genes. [file 12943_2015_432_MOESM2_ESM.pdf]
