# Supplementary material for: Knockdown of NAT12/NAA30 reduces tumorigenic features of glioblastoma-initiating cells
Source: Mol Cancer. 2015 Aug 21;14:160. doi: 10.1186/s12943-015-0432-z (PMC4546247; doi:10.1186/s12943-015-0432-z)
Supplement: Additional file 3: Supplementary File 1. — Oligonucleotides and microarray probes. (PDF 115 kb) [file 12943_2015_432_MOESM3_ESM.pdf]

Supplementary file 1

Probes and oligonucleotides used for expression analysis of *NAA30/NAT12*

Microarray probes:

1. **ILMN_2128087 (used in this study)**
2. AGCCGGGAATTGCTGGGGTTTAGATGCACTTTTTCTTTTGAGAGTAAGGG and mapped to the 2085-2134 region (3ÚTR) of the reference requence: NM_001011713.2.
3. **Affimetrix 225679_at** (REMBRANDT database) corresponds to the 3'UTR 4005-4404 of the reference sequence NAT12/NAA30, NM_001011713.2.
4. **Affimetrix 228322_at** (REMBRANDT database) correspond to the coding region 581-877 of the reference sequence of the NAT12/NAA30, NM_001011713.2.
5. **Affimetrix 228321_at** (REMBRANDT database) correspond to the coding region 944-1061of the reference sequence of the NAT12/NAA30, NM_001011713.2.

Oligonucleotides:

>Oli_79_DIR_RPL30_228; AGCTTTGAGGAAATCTGAAATAGAGTA
>Oli_80_REV_RPL30_441; GTGAAATTTTGTAGGTGAAAAGGTTTA
>Oli_81_DIR_RPL27_226; GTATAACTACAATCACCTAATGCCCAC
>Oli_82_REV_RPL27_477; TTTAATGATCAAAACAAAGCATCTAAA
>Oli_117_DIR_GFAP_V2_set1_1523; AGTAACTGTACATTAAACTGGCAGAGC
>Oli_118_REV_GFAP_V2_set1_1817; TAACATTAAGAGCAGGGAACATAAAAC
>Oli_123_DIR_GFAP_V1_set2_2101; GATGACTCAAGTGTCTCAGTCCAC
>Oli_124_REV_GFAP_V1_set2_2434; GAGTCACTTCCTTAATTCCCACAAT
>Oli_129_DIR_NES_set1_5247; CTAAGTCAGCTGAATCCCGATAGTA
>Oli_130_REV_NES_set1_5544; CATTACTTTATTCAGGCAGGGACTA
>Oli_135_DIR_TUB_set1_1368; AGATGTACGAAGACGACGAGGAG
>Oli_136_REV_TUB_set1_1712; TTAAGGGTATCTGACAGCAATAGATTT
>Oli_279_DIR_NAT12_setG_4086; CTGACATGTCTAAGACTGGATGTGTAT
>Oli_280_REV_NAT12_setG_4412; CATTAAATCTCTCTCCAAAAGGATATG
>Oli_281_DIR_NAT12_setF_3579; TTTTGATAATAGGCTGTTGTTTTTCTT
>Oli_282_REV_NAT12_setF_3840; ACACATCAAAAATGATAGTATACCCGT
>Oli_283_DIR_NAT12_setE_2632; ACTGTAGGAATCTGTTGTTGTTTTCAT
>Oli_284_REV_NAT12_setE_2843; CACTTTCAAAACACTATACTGAGCAAA
>Oli_285_DIR_NAT12_setD_1328; GATTTAGTAATTTCCATGCAGCTCTTA
>Oli_286_REV_NAT12_setD_1571; TATGTTCCACTTACTGGTTCTGTGATA
>Oli_287_DIR_NAT12_setC_1061; TTAAGAAAGCTATATATGCCATGGTTG
>Oli_288_REV_NAT12_setC_1268; GATAGTTGTTCCTTGATGTCAGTTTCT
>Oli_289_DIR_NAT12_setB_572; ACTCCCTCTCTAGTAATGCAAGAACT
>Oli_290_REV_NAT12_setB_886; TATAGGTATAAATGGAGTAGGGTTCGG
>Oli_291_DIR_NAT12_setA_398; AGCAGCTCAACGGATTGATTAG
>Oli_292_REV_NAT12_setA_597; AGTTCTTGCATTACTAGAGAGGGAGT
