# Supplementary material for: Knockdown of NAT12/NAA30 reduces tumorigenic features of glioblastoma-initiating cells
Source: Mol Cancer. 2015 Aug 21;14:160. doi: 10.1186/s12943-015-0432-z (PMC4546247; doi:10.1186/s12943-015-0432-z)
Supplement: Additional file 4: Supplemetary File 2. — Additional qPCR statistics for Fig. 1, Fig. 3 and Fig. 5. (PDF 254 kb) [file 12943_2015_432_MOESM4_ESM.pdf]

Supplementary file 2

Additional qPCR statistics for Figure 1

Expression of NAT12/NAA30 in GICs compared to NSCs (Figure 1D)

Relative Expression Results

Gene	Expression	Std. Error	95% C.I.	P(H1)	Result
G	0.034	0,008 - 0,508	0,004 - 1,166	0	DOWN
F	0.563	0,323 - 0,960	0,180 - 1,354	0.012	DOWN
E	0.456	0,213 - 1,099	0,095 - 1,209	0.007	DOWN
D	0.634	0,215 - 1,938	0,019 - 3,427	0.268	
C	0.472	0,222 - 1,366	0,020 - 1,802	0.033	DOWN
B	0.785	0,379 - 1,926	0,052 - 3,415	0.532	
A	0.826	0,372 - 1,712	0,143 - 17,279	0.641	

Expression of NAT12/NAA30 in GICs compared to NSCs (Figure 1F)

Gene	Expression	Std. Error	95% C.I.	P(H1)	Result
RPL30	1				
ex 2-3	2.114	0,269 - 9,983	0,109 - 168,848	0.266	
ex 3-4	1.03	0,293 - 4,919	0,001 - 27,665	0.97	
ex 4-5	1.268	0,256 - 4,506	0,109 - 18,422	0.672	

Additional qPCR statistics for Figure 3

Relative expression of NAT12/NAA30 in KD1 (Figure 3A)

	KD1					
	Gene	RE	Std. Error	95% C.I.	P(H1)	Result
	RPL30	1				
NAT12/NAA30	A (398-597)	0.748	0,386 - 1,808	0,309 - 1,980	0.35	
	B (572-886)	0.501	0,278 - 0,745	0,228 - 1,126	0.011	DOWN
	C (1061-1268)	0.301	0,203 - 0,452	0,171 - 0,586	0.001	DOWN
	D (1328-1571)	1.294	0,836 - 2,116	0,808 - 2,565	0.146	
	E (2632-2843)	0.564	0,380 - 0,940	0,363 - 0,969	0.001	DOWN
	F (3579-3840)	0.978	0,426 - 1,711	0,351 - 1,982	0.915	
	G (4086-4412)	0.829	0,486 - 1,575	0,362 - 1,606	0.367	

Relative expression of NAT12/NAA30 in KD2 (Figure 3B)

	KD2					
	Gene	RE	Std. Error	95% C.I.	P(H1)	Result
	RPL30	1				
NAT12/NAA30	A (398-597)	0.683	0,430 - 1,210	0,367 - 2,420	0.368	
	B (572-886)	0.466	0,342 - 0,649	0,294 - 0,752	0.002	DOWN
	C (1061-1268)	0.422	0,266 - 0,689	0,195 - 0,873	0.019	DOWN
	D (1328-1571)	0.719	0,554 - 0,928	0,461 - 1,321	0.099	
	E (2632-2843)	0.764	0,477 - 1,087	0,368 - 1,236	0.256	
	F (3579-3840)	0.761	0,566 - 1,101	0,428 - 1,218	0.167	
	G (4086-4412)	0.85	0,515 - 1,179	0,420 - 1,421	0.61	

Additional qPCR statistics for Figure 5

Relative Expression of GFAP, NES and TUBIII in NAT12/NAA30 KD1 and KD2 (Figure 5B)

Gene	RE	Std. Error	95% C.I.	P(H1)	Result
RPL30	1				
GFAP1	55.637	32,739 - 103,666	23,247 - 236,915	0	UP
GFAP2	21.545	13,304 - 31,331	11,388 - 72,955	0	UP
NESTIN	0.981	0,692 - 1,309	0,575 - 2,863	0.926	
TUBULINIII	1.183	0,817 - 1,556	0,644 - 3,336	0.348	
