# Supplementary material for: Knockdown of NAT12/NAA30 reduces tumorigenic features of glioblastoma-initiating cells
Source: Mol Cancer. 2015 Aug 21;14:160. doi: 10.1186/s12943-015-0432-z (PMC4546247; doi:10.1186/s12943-015-0432-z)
Supplement: Additional file 5: Figure S3. — Kaplan-Meier survival plot for NAT genes (NAT12, NAT1, NAT2 and NAT10). P values (Log-rank test) signify the differences in patient survival in the selected groups with the following parameters: Up-Regulated: fold ≥ 2; Down-Regulated: fold ≥ 2. Reporter Type: Affymetrix. [file 12943_2015_432_MOESM5_ESM.pdf]

Supplementary Figure 3

A

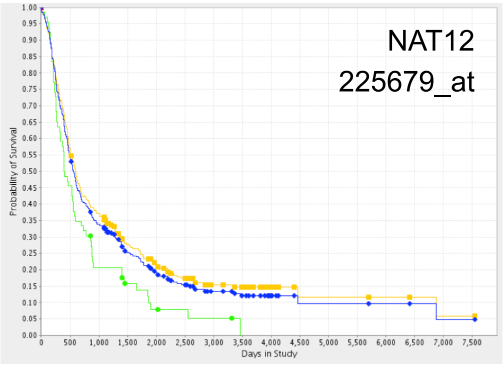

Number of samples in group:
Down-Regulated: 66
Intermediate: 277
Up-Regulated vs. Intermediate: p= 0.0060614742

B

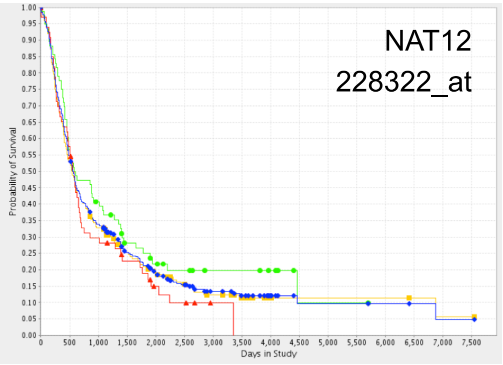

Number of samples in group:
Up-Regulated: 66
Down-Regulated: 76
Intermediate: 201
Up-Regulated vs. Intermediate: p= 0.5535712293
Down-Regulated vs. Intermediate: p= 0.2266796943

C

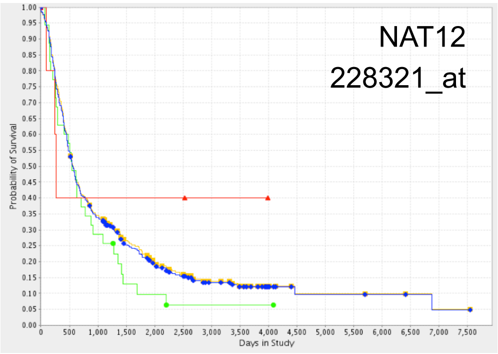

Number of samples in group:
Up-Regulated: 5
Down-Regulated: 35
Intermediate: 303
Up-Regulated vs. Intermediate: p= 0.5067904872
Down-Regulated vs. Intermediate: p= 0.2177514054

D

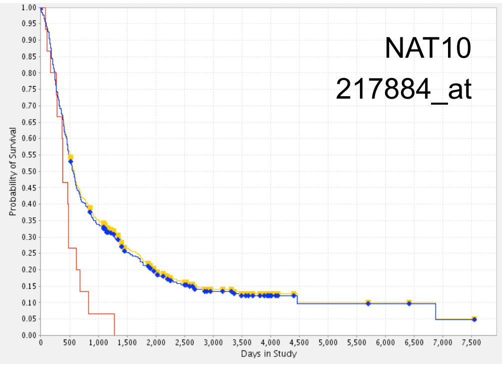

Number of samples in group:
Up-Regulated: 15
Intermediate: 328
Up-Regulated vs. Intermediate: p= 0.0060838757

E

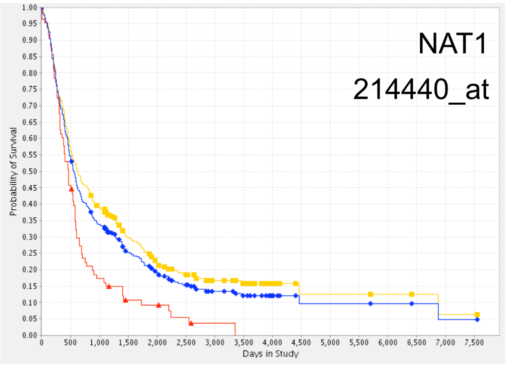

Number of samples in group:
Up-Regulated: 83
Intermediate: 260
Up-Regulated vs. Intermediate: p= 1.130635E-4

F

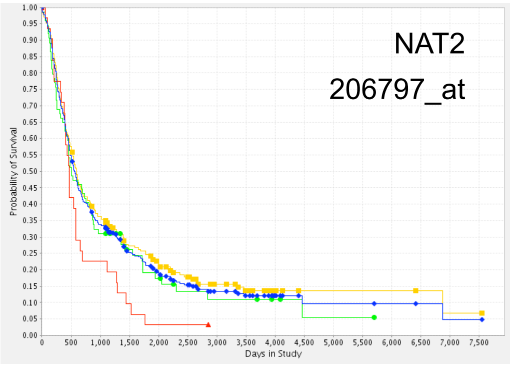

Number of samples in group:
Up-Regulated: 31
Intermediate: 238
Up-Regulated vs. Intermediate: p= 0.02067296
