# Supplementary material for: Knockdown of NAT12/NAA30 reduces tumorigenic features of glioblastoma-initiating cells
Source: Mol Cancer. 2015 Aug 21;14:160. doi: 10.1186/s12943-015-0432-z (PMC4546247; doi:10.1186/s12943-015-0432-z)

Supplementary Figure 5

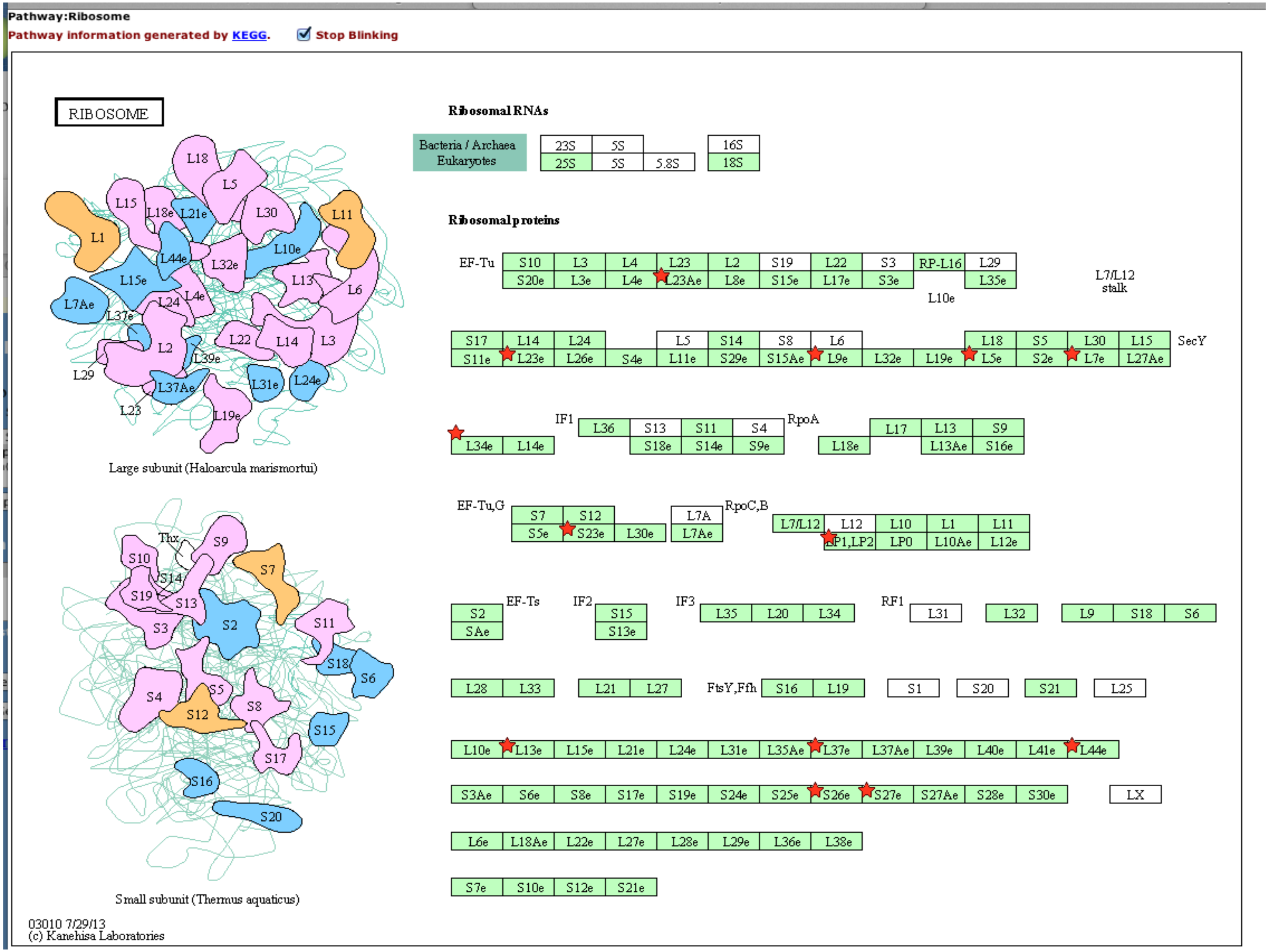

Supplement: Additional file 8: Figure S5-S7. — Figure S5. NAT12/NAA30 knockdown resulted in dysregulation of ribosome assembly as shown by microarray analysis. Using the DAVID functional annotation tool we analyzed the KEGG pathway “Ribosome” where 13 genes (p=5.6E-06) were differentially regulated in KD1 and KD2. See also Supplementary File 3. Dysregulated genes are marked with red asterisks. Figure S6. NAT12/NAA30 knockdown resulted in dysregulation of the p53 pathway as shown by microarray analysis. Using the DAVID functional annotation tool we analyzed the KEGG pathway “p53” where 8 genes (p=3.1E-03) were differentially regulated in KD1 and KD2. See also Supplementary File 3. Dysregulated genes are marked with red asterisks. Figure S7. NAT12/NAA30 knockdown resulted in dysregulation of sphingolipid metabolism as shown by microarray analysis. Using the DAVID functional annotation tool we analyzed the KEGG pathway “Sphingolipid metabolism” where 6 genes (p=4.8E-03) were differentially regulated in KD1 and KD2. See also Supplementary File 3. [file 12943_2015_432_MOESM8_ESM.zip › add8/1018244584147474_add4.pdf]
