# Supplementary material for: Knockdown of NAT12/NAA30 reduces tumorigenic features of glioblastoma-initiating cells
Source: Mol Cancer. 2015 Aug 21;14:160. doi: 10.1186/s12943-015-0432-z (PMC4546247; doi:10.1186/s12943-015-0432-z)
Supplement: Additional file 12: Supplementary File 4. — List of antibodies used for western blot. (DOCX 53 kb) [file 12943_2015_432_MOESM12_ESM.docx]

**Supplementary file 4**

**List of antibodies**

| **PRIMARY ANTIBODIES-WESTERN** | | | | | |
| --- | --- | --- | --- | --- | --- |
|  | **NAME OF THE ANTIBODY** | **PRODUCT NUMBER** | **COMPANY** | **ANIMAL** | **DILUTION** |
| 1 | ACTIN | #4967 | Cell Signaling Technology | rabbit | 1:1000 |
| 2 | GLI1 | #2534 | Cell Signaling Technology | rabbit | 1:1000 |
| 3 | GFAP (GA5) | #3670 | Cell Signaling Technology | mouse | 1:1000 |
| 4 | NAT12 | **HPA057824** | Sigma-Aldrich | rabbit | 1:250 |
| 5 | NAT12-C terminal | SAB1303349 | Sigma-Aldrich | rabbit | 1:250 |
| 6 | p-p70 S6 kinase (Thr 389) | #9205 | Cell Signaling Technology | rabbit | 1:1000 |
| 7 | p53 | #2524 | Cell Signaling Technology | rabbit | 1:1000 |
| 8 | p-p53 (Ser 37) | #9289 | Cell Signaling Technology | rabbit | 1:1000 |
| 9 | p-p53 (Ser 15) | #9284 | Cell Signaling Technology | rabbit | 1:1000 |
| 10 | p-mTOR (Ser 2448) | 5536P | Cell Signaling Technology | rabbit | 1:1000 |
| 11 | mTOR | #2983 | Cell Signaling Technology | rabbit | 1:1000 |
| 12 | pSTAT3(Ser727) | SAB300034 | Sigma-Aldrich | rabbit | 1:1000 |
| 13 | SHH (N-term) | MAB464 | RD Systems | rat | 1:500 |
| 14 | ABC Active b-Catenin | #05-665 | Millipore | mouse | 1:500 |
| 15 | KCNMA1 | Ab99046 | Abcam | rabbit | 1:1000 |
| 16 | CCND1 | #2922 | Cell Signaling Technology | rabit | 1:1000 |
| 17 | pAKT(Ser473) | #9271 | Cell Signaling Technology | rabbit | 1:1000 |
| 18 | pAKT(Thr308) | #9275 | Cell Signaling Technology | rabbit | 1:1000 |
| 19 | p-p4E-BP1 (Thr37/46) | #2855 | Cell Signaling Technology | rabitt | 1:1000 |
| 20 | SOX2 | AF2018 | R&D | goat | 1:1000 |
| 21 | NESTIN | MAB5326 | Millipore | mouse | 1:1000 |
| 22 | HIF1α | #14179 | Cell Signaling Technology | rabbit | 1:1000 |
|  |  |  |  |  |  |
|  |  |  |  |  |  |
| **SECONDARY ANTIBODIES-WESTERN** | | | | | |
|  | **NAME OF THE ANTIBODY** | **PRODUCT NUMBER** | **COMPANY** | **ANIMAL** | **DILUTION** |
| 1 | ECL Anti-rabbit IgG-HRP | NA934 | Amersham | donkey | 1:10000 |
| 2 | ECL Anti-mouse IgG-HRP | NA931 | Amersham | sheep | 1:10000 |
| 3 | Anti-mouse IgG-HPR | #7076 | Cell Signaling Technology | horse | 1:10000 |
| 4 | Anti-rat IgG | NA935 | Amersham | goat | 1:10000 |
